# MdWRKY31‐MdNAC7 regulatory network: orchestrating fruit softening by modulating cell wall‐modifying enzyme MdXTH2 in response to ethylene signalling

**DOI:** 10.1111/pbi.14445

**Published:** 2024-08-23

**Authors:** Jia‐Hui Wang, Quan Sun, Chang‐Ning Ma, Meng‐Meng Wei, Chu‐Kun Wang, Yu‐Wen Zhao, Wen‐Yan Wang, Da‐Gang Hu

**Affiliations:** ^1^ National Research Center for Apple Engineering and Technology, Shandong Collaborative Innovation Center of Fruit & Vegetable Quality and Efficient Production, College of Horticultural Science and Engineering Shandong Agricultural University Tai'an Shandong China; ^2^ College of Horticulture Agricultural University of Hebei Baoding Hebei China

**Keywords:** apple, ethylene, fruit softening, MdWRKY31, MdNAC7, MdXTH2

## Abstract

Softening in fruit adversely impacts their edible quality and commercial value, leading to substantial economic losses during fruit ripening, long‐term storage, long‐distance transportation, and marketing. As the apple fruit demonstrates climacteric respiration, its firmness decreases with increasing ethylene release rate during fruit ripening and postharvest storage. However, the molecular mechanisms underlying ethylene‐mediated regulation of fruit softening in apple remain poorly understood. In this study, we identified a WRKY transcription factor (TF) MdWRKY31, which is repressed by ethylene treatment. Using transgenic approaches, we found that overexpression of MdWRKY31 delays softening by negatively regulating xyloglucan endotransglucosylase/hydrolases 2 (*MdXTH2*) expression. Yeast one‐hybrid (Y1H), electrophoretic mobility shift (EMSA), and dual‐luciferase assays further suggested that MdWRKY31 directly binds to the *MdXTH2* promoter via a W‐box element and represses its transcription. Transient overexpression of ethylene‐induced *MdNAC7*, a NAC TF, in apple fruit promoted softening by decreasing cellulose content and increasing water‐soluble pectin content in fruit. MdNAC7 interacted with MdWRKY31 to form a protein complex, and their interaction decreased the transcriptional repression of MdWRKY31 on *MdXTH2*. Furthermore, MdNAC7 does not directly regulate *MdXTH2* expression, but the protein complex formed with MdWRKY31 hinders MdWRKY31 from binding to the promoter of *MdXTH2*. Our findings underscore the significance of the regulatory complex NAC7–WRKY31 in ethylene‐responsive signalling, connecting the ethylene signal to *XTH2* expression to promote fruit softening. This sheds light on the intricate mechanisms governing apple fruit firmness and opens avenues for enhancing fruit quality and reducing economic losses associated with softening.

## Introduction

Apple (*Malus domestica* Borkh.), one of the most widely produced and economically important fruit crops in temperate regions of the world, is rich in minerals and vitamins (Duan *et al*., 2017). Fruit softening is a pivotal process that governs the physiological maturation of various fruit. However, this phenomenon presents a significant challenge as it adversely affects the edible quality and commercial value of fruit, leading to economic losses during long‐term storage and long‐distance marketing (Win *et al*., 2019).

Fruit softening constitutes complex physiological and biochemical processes, often modulated by multiple polysaccharide‐degrading enzymes (Peng *et al*., 2022). Notably, the plant cell wall comprising cellulose, hemicellulose, polysaccharides, and a limited amount of structural proteins serves as a characteristic feature that distinguishes it from animal cell wall (Somerville *et al*., 2004). Fruit softening primarily results from modifications in cell wall structure and the degradation of its components. Moreover, cell wall metabolism is closely related to ripening‐associated changes in structure and texture. Key alterations in the cell wall during ripening involve depolymerization of pectin and hemicellulose and pectinolytic activity, leading to the dissolution of the intermediate layer, reduced cell adhesion, and cell wall swelling, rendering the substrate more vulnerable to enzymatic action (Tucker *et al*., 2017; Wang *et al*., 2018).

This process of fruit softening involves the coordinated action of multiple cell wall modification enzymes, including cellulase, polygalacturonase (PG), β‐galactosidase (β‐gal), pectate lyase (PL), and xyloglucan endotransferase/hydrolase (XTH) (Belleau‐Deytieux *et al*., 2009; Opazo *et al*., 2013; Qian *et al*., 2016; Tucker *et al*., 2017; Wang *et al*., 2018). β‐gal plays a critical role in increasing cell wall permeability by depolymerizing the galactose side chains of xyloglucan, rhamnogalacturonan, and hemicellulose, thereby enabling cell wall hydrolases such as PG to interact with pectin and accelerate fruit softening (Gerardi *et al*., 2012; Posé *et al*., 2013). XTH belongs to a subgroup of the glycoside hydrolase family 16 (GH16) of the Carbohydrate Active Enzyme Family Database (CAZy). XTH proteins contain a Glyco_hydro_16 protein domain and a XET_C domain (Behar *et al*., 2018; Eklöf and Brumer, 2010). XTHs participate in xyloglucan metabolism, thereby exhibiting xyloglucan endoglycosylase (XET) or xyloglucan endohydrolase (XEH) activity (Saladié *et al*., 2006). XTHs play a vital role in fruit ripening and softening, as they facilitate cell wall loosening and cellulose–xyloglucan breakdown (Cosgrove, 2005). Although the biological process of softening has been well investigated, the molecular regulatory mechanism is still unknown.

Fruit softening occurs during fruit ripening and is regulated by several transcription factors (TFs) related to fruit softening. There is also growing evidence that the NAC and WRKY TFs are involved in regulating fruit softening. For instance, inhibiting *SNAC4* in tomatoes prevents the accumulation of ABA and maintains the firmness during fruit ripening (Yang *et al*., 2021). Another NAC TF, *SlNAC1*, is overexpressed in tomatoes, which results in reduced fruit firmness and thinner peels than in wild‐types (Ma *et al*., 2014). Through MaXB3‐mediated ubiquitination degradation pathways, *MaNAC1* and *MaNAC2* negatively regulate the ethylene synthesis of banana fruit, resulting in the inhibition of the softening (Shan *et al*., 2020). The ethylene treatment of apple fruit postharvest induced MdMAPK3 phosphorylation of MdNAC72, which then prompted MdPUB24 to ubiquitin MdNAC72 and cause its subsequent destruction, accelerating the process of apple fruit postharvest storage softening (Wei *et al*., 2023). Other studies have shown that WRKY TFs participate in plant softening. Over the past two decades, considerable research has focused on the biological functions of WRKY TFs, particularly in plant responses to various stresses, including biotic and abiotic stresses. While WRKY TFs were initially considered resistance proteins, recent discoveries have highlighted their essential roles in the softening during fruit ripening. In strawberries, FvWRKY48 influences fruit softening by promoting *FvPLA* expression, leading to pectin degradation (Zhang *et al*., 2022). Beyond their role in fruit softening, recent findings from our study demonstrate that MdWRKY31 in apple interacts with MdERF72 to regulate *MdALMT9* expression, thus impacting postharvest fruit acidity (Wang *et al*., 2023b). In apples, MdHY5 interacts with MdWRKY31, suppressing its expression and inhibiting MdWRKY31–MdLAC7 interaction to regulate apple browning (Wang *et al*., 2023a). Meng *et al*. (2016) found that *MdWRKY31* was highly expressed in the shoot tip, leaf, fruit, and callus of apple. Given that WRKY TFs constitute one of the largest regulatory protein families in plants, their role as major regulators of fruit quality is evident. However, the function of NAC and WRKY as well as their interaction in regulating apple fruit softening is not well understood.

A series of factors, including hormones, temperature, and gas composition, influence fruit softening (Barka *et al*., 2000; Shi *et al*., 2022), and of these, ethylene generally has the greatest effect (Bu *et al*., 2013; Harb *et al*., 2012; Tatsuki *et al*., 2013). Notably, the expression of fruit softening‐related genes *PpGAL1* and *PpGAL4* in late‐ocean pears is influenced by exogenous ethylene (upregulated) or 1‐MCP (downregulated) (Mwaniki *et al*., 2005). Similarly, the expression of *DkGAL1* implicated in persimmon fruit softening is also regulated by ethylene (Ban *et al*., 2016). However, the specific molecular mechanism by which ethylene regulates fruit softening is not clear in apple. Through our current investigation, we determined that MdWRKY31 interacts with MdNAC7, leading to the alleviation of transcriptional repression of *MdXTH2* by MdWRKY31. Consequently, this regulatory interaction contributes to fruit softening by loosening the cell wall and facilitating catabolic activities within the cellulose–xyloglucan matrix. This discovery culminates in the establishment of an MdWRKY31–MdNAC7–MdXTH2 regulatory network unravelling the molecular mechanisms through which ethylene regulates fruit softening.

## Results

### *MdWRKY31* overexpression significantly increases cell wall strength in apple

**Figure 1:**
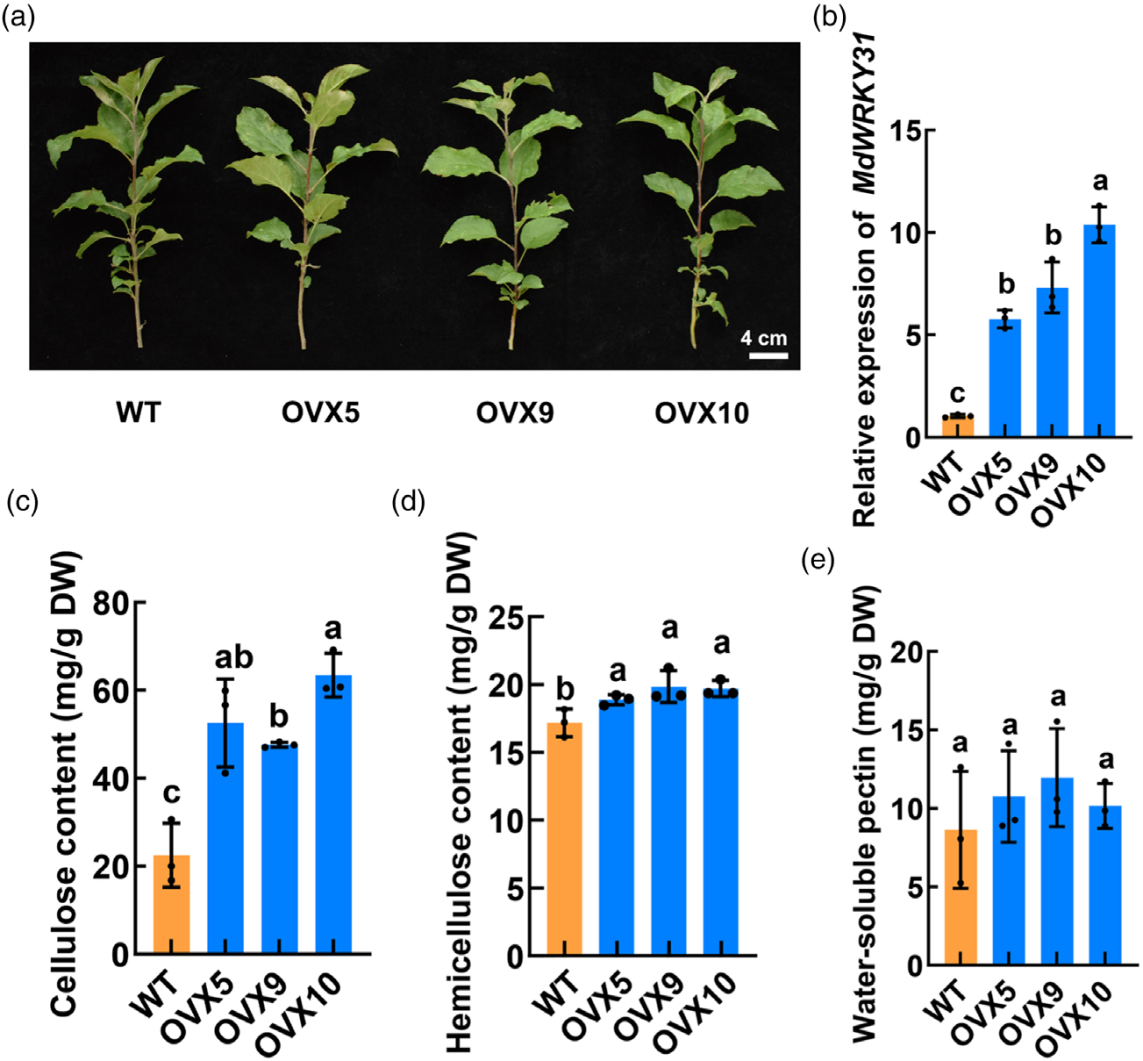
MdWRKY31 positively contributes to cell wall strength in apple. (a) Three MdWRKY31‐OVX transgenic apple lines and wild‐type (WT). Scale bar = 4 cm. (b) Relative expression level of *MdWRKY31* in the three MdWRKY31‐OVX lines and WT. (c–e) Cellulose content (c), hemicellulose content (d), and water‐soluble pectin content (e) in MdWRKY31‐OVX transgenic apple lines and WT. Data are shown as the mean ± SD. Experiments were repeated three biological replicates. Different letters above the columns indicate significant differences (*P* < 0.05) as determined by one‐way ANOVA.

In our previous study, we successfully generated three stable lines with overexpressed *MdWRKY31* in apple plants (Wang *et al*., 2023b). Due to the extended juvenile period (6–8 years) in apple plants, which are perennial woody plants, after germination and before flowering and fruit set, fruit quality evaluation during this phase is not feasible. Consequently, we directed our focus towards investigating the impact of *MdWRKY31* overexpression on cell wall components in the leaves of wild‐type (WT) and *WRKY31* overexpression apple plants. Through comprehensive analysis of cellulose, hemicellulose, and soluble pectin contents in the leaves of the three stable *MdWRKY31* overexpression lines and the WT (Figure 1a,b), intriguing results emerged. Specifically, compared with the WT, the *MdWRKY31* overexpression apple plants exhibited a significant increase in cellulose and hemicellulose contents (Figure 1c,d). Conversely, no discernible difference was observed in the soluble pectin content (Figure 1e). These findings imply that MdWRKY31 positively contributes to cell wall strength in apple.

### *MdWRKY31* overexpression enhances fruit firmness

**Figure 2:**
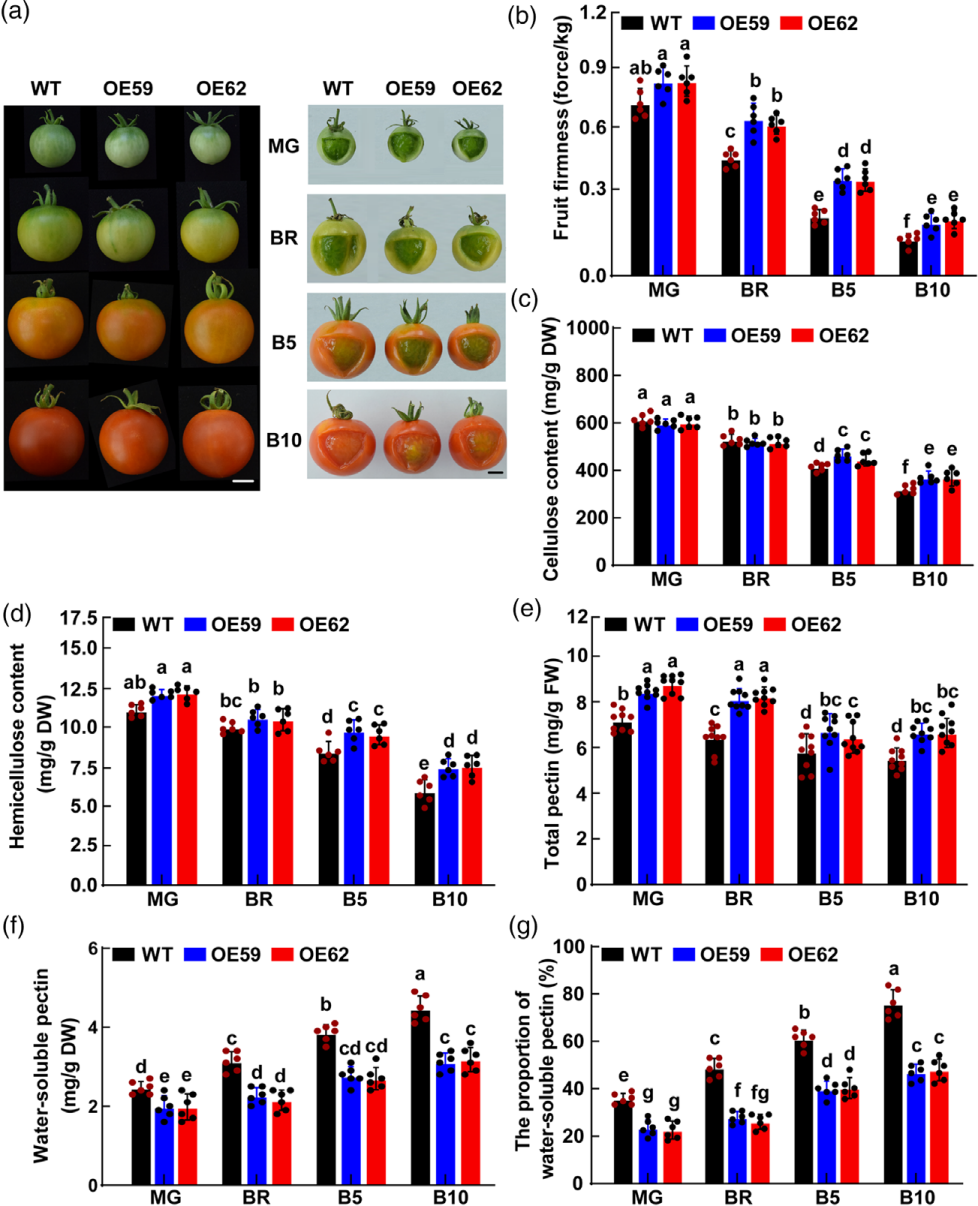
Overexpression of *MdWRKY31* improves tomato fruit firmness during fruit ripening. (a) WT and MdWRKY31‐OEs (OE59 and OE62) tomato fruit at mature green (MG), breaker stage (BR), breaker+5d (B5), and breaker+10d (B10). Scale bars represent 1 cm. (b) WT and MdWRKY31‐OEs tomato fruit at indicated developmental stages with pericarp partially removed to see locule development. Scale bars represent 15 mm. (b–g) Fruit firmness (b), cellulose content (c), hemicellulose content (d), total pectin (e), water‐soluble pectin content (f), and the proportion of water‐soluble pectin (g) of tomato fruit in WT and two *MdWRKY31* transgenic lines at MG, BR, B5, and B10 stages. Data are shown as the mean ± SD. Experiments were repeated nine independent times. Different letters above the columns indicate significant differences (*P* < 0.05) as determined by one‐way ANOVA.

To explore the effect of MdWRKY31 on fruit softening, we first conducted heterologous overexpression of the *MdWRKY31* gene in tomato, resulting in the establishment of two tomato lines with stable *MdWRKY31* overexpression (Figure 2a). Subsequently, we evaluated various softening characteristics, including fruit firmness, the content of cellulose, hemicellulose, total pectin, and water‐soluble pectin, as well as the proportion of water‐soluble pectin at different ripening stages: mature green stage (MG, ~39 days after flowering), breaker stage (BR, when fruit change from green to yellow‐brown, ~42 days after flowering), B5 (light red, approximately breaker +5 days), and B10 (full red stage, approximately breaker +10 days). Our observations revealed distinct differences between the tomato fruit with stable overexpression of *MdWRKY31* and the WT fruit at the same stages. The tomato fruit overexpressing *MdWRKY31* presented higher firmness than that of WT during the ripening process, except for the MG stage (Figure 2b). A gradual decline in the cellulose and hemicellulose contents was noted from the green ripening stage to the full red stage in both groups (Figure 2c,d). Only at B5 and B10 stages, the cellulose and hemicellulose contents in *MdWRKY31*‐overexpressing fruit were higher than those in WT fruit (Figure 2c,d). Notably, the total pectin content in *MdWRKY31*‐overexpressing fruit was significantly higher than that in WT throughout the ripening period (Figure 2e). On the contrary, the water‐soluble pectin and the proportion of water‐soluble pectin in *MdWRKY31*‐overexpressing fruit were significantly higher than those in WT along with tomato fruit ripening (Figure 2f,g). These results indicate that *MdWRKY31* overexpression enhances fruit firmness during tomato fruit ripening.

**Figure 3:**
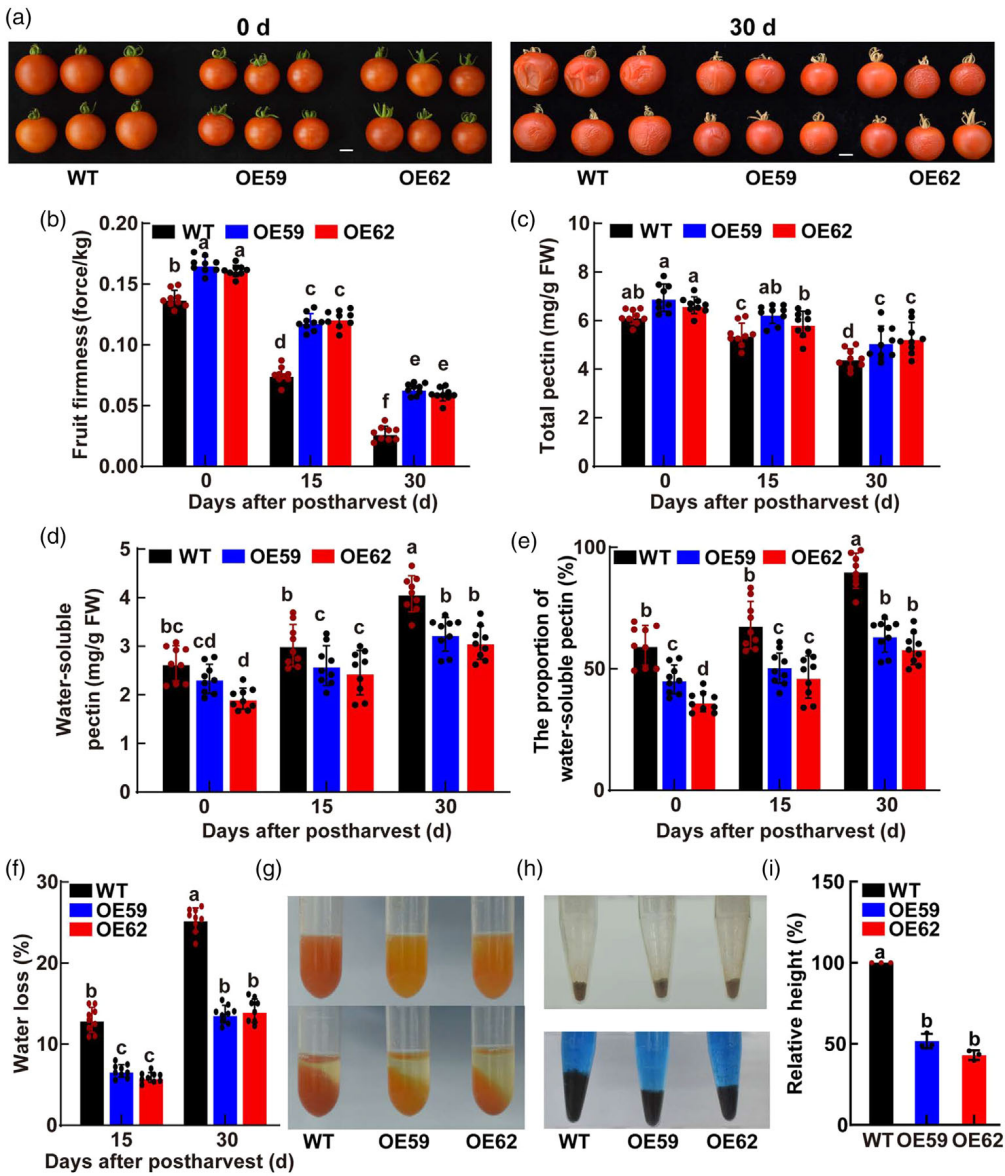
Overexpression of *MdWRKY31* maintains tomato fruit firmness during postharvest. (a) WT and MdWRKY31‐OEs (OE59 and OE62) tomato fruit during postharvest. Scale bars represent 1 cm. Fruit firmness (b), total pectin (c), water‐soluble pectin content (d), and the proportion of water‐soluble pectin (e), water loss (f) of tomato fruit in WT and two *MdWRKY31* transgenic lines during postharvest. Data are shown as the mean ± SD. Experiments were repeated nine independent times. Different letters above the columns indicate significant differences (*P* < 0.05) as determined by one‐way ANOVA. Gel tissue (g) and cell wall expansion (h) of tomato fruit in WT and two *MdWRKY31* transgenic lines at B10 stage. (i) The relative height of the settled sediment was taken as a measure of cell wall swelling. Data are shown as the mean ± SD. Every experiment was performed at least in triplicate. Different letters above the columns indicate significant differences (*P* < 0.05) as determined by one‐way ANOVA.

To comprehensively evaluate the function of *MdWRKY31* in regulating fruit firmness, we further measured the fruit firmness and cell wall‐related components of WT and *MdWRKY31* transgenic tomato fruit during postharvest storage (Figure 3). As shown in Figure 3a, after 30 days of postharvest storage, WT fruit showed obvious symptoms of fruit collapse, denting, and wilting, while *MdWRKY31*‐overexpressing fruit remained plump and intact. Further analysis found that the fruit firmness and total pectin content of *MdWRKY31*‐overexpressing fruit were significantly higher than that of WT (Figure 3b,c), but the water‐soluble pectin, the proportion of water‐soluble pectin and the fruit water loss rate was significantly lower than that of WT during the whole storage process (Figure 3d–f). In addition, compared with WT fruit, *MdWRKY31*‐overexpressing fruit exhibited reduced gel liquefaction, and their gel tissue released moisture more easily (Figure 3g), moreover, the cell wall expansion in these genetically modified tomatoes decreased by nearly 50% compared with that of WT (Figure 3h,i). These results are consistent with the transgenic tomatoes that exhibit a lower rate of water loss and a higher fruit firmness compared with the control (Figure 3b,f), leading to an extended shelf life. In addition, we determined the cuticular wax crystals using scanning electron microscopy (SEM) as well as cuticular total wax content and components using gas chromatography–mass spectrometry (GC–MS) to determine the changes in cuticular wax in tomato fruits during postharvest storage (Figure S1), but neither showed a difference between WT and *MdWRKY31*‐overexpressing fruit, suggesting that the significant differences in fruit firmness and water loss between *MdWRKY31*‐overexpressing fruit and WT were due to the regulation of cell wall degradation and modification by MdWRKY31 rather than the effect on the cuticle.

Collectively, our findings further underscore the substantial role of MdWRKY31 in enhancing fruit firmness during fruit ripening and postharvest storage.

### Overexpression of *MdWRKY31* affects the expression of cell wall‐modifying enzymes

**Figure 4:**
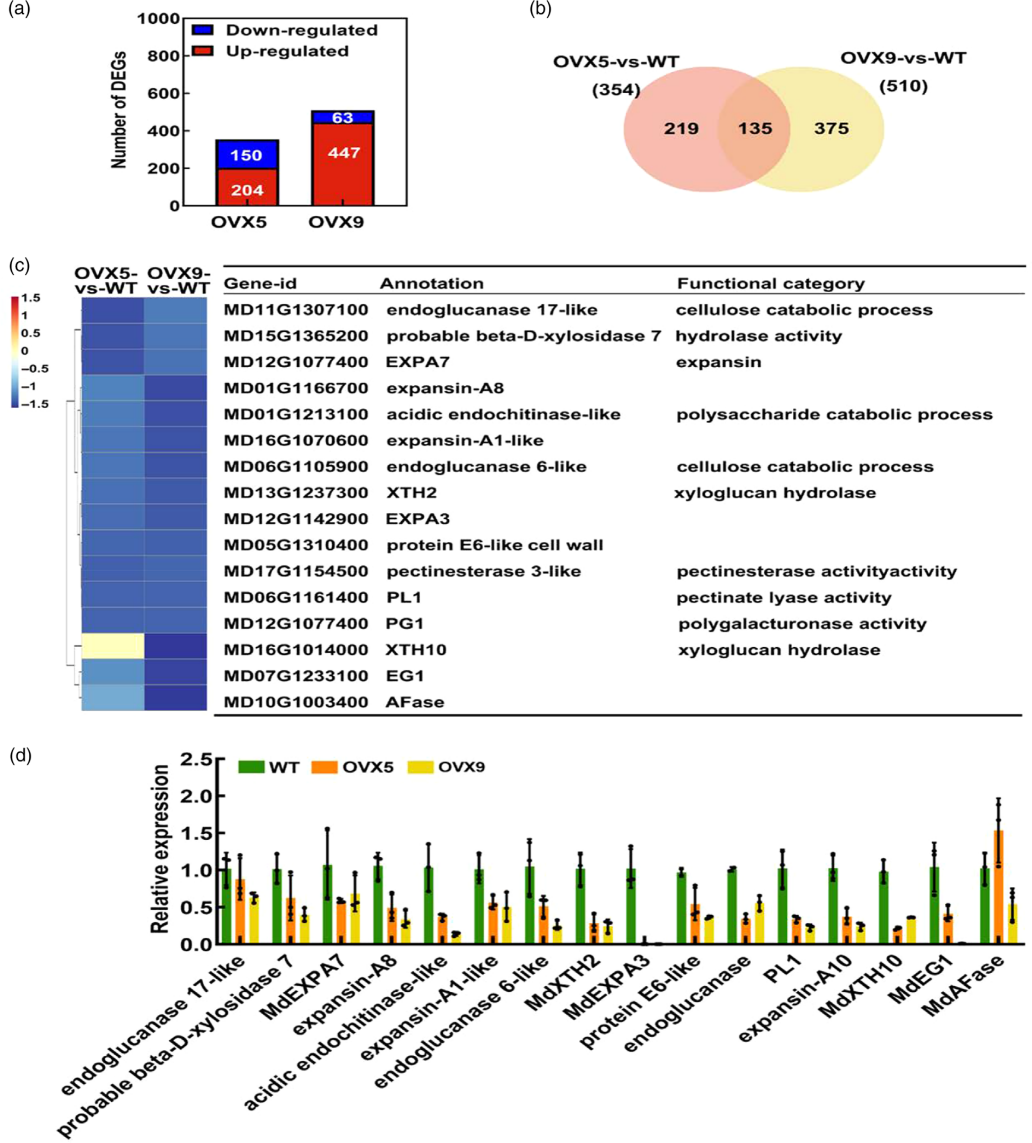
Overexpression of *MdWRKY31* affects the expression of cell wall‐modifying enzymes. (a) Number of differentially expressed genes (DEGs) in the biological processes of the two *MdWRKY31* transgenic apple lines. (b) Venn diagram of differentially expressed genes co‐regulated in the two *MdWRKY31* transgenic apple lines. (c) Subset of the 16 cell wall–associated DEGs, annotation, functional categories, and expression (log 2 of reads per kilobase million). (d) Expression of genes involved in cell wall modification in WT and *MdWRKY31* overexpressed apple leaves. The experiments were performed in three technical replicates with two technical repetitions for each biological sample. Error bars indicate ± SD.

To investigate the molecular mechanism underlying the regulation of fruit firmness by *MdWRKY31*, we conducted RNA‐seq analysis using both WT and *MdWRKY31* transgenic apple plants. In the *MdWRKY31* overexpression lines (OVX5 and OVX9), we identified a total of 354 (204 were upregulated and 150 were downregulated) and 510 (447 were upregulated and 63 downregulated) differentially expressed genes, respectively (Figure 4a). Among these, 135 differentially expressed genes were found to be shared between the OVX5 and OVX9 lines (Figure 4b). Upon analysis of the 135 differentially expressed genes, we observed a significant suppression of genes involved in cell wall modification in the *MdWRKY31* transgenic strain (Figure 4c). A reverse transcription quantitative PCR (RT‐qPCR) analysis further confirmed the RNA‐seq data (Figure 4d). Overall, our comprehensive analyses demonstrate that MdWRKY31 negatively regulates the expression of cell wall‐modifying enzymes in apple.

To further substantiate the regulatory role of *MdWRKY31* in fruit firmness through modulation of the expression of cell wall‐modifying enzymes, we conducted RT‐qPCR analysis of previously reported cell wall modification‐related genes (*SlCEL2*, *SlEXP1*, *SlPG2a*, *SlXTH5*, and *SlPL1*) known to regulate fruit firmness in tomato. As the fruit ripened, we noted a gradual decrease in the expression levels of cell wall‐modifying enzymes after the BR stage. However, intriguingly, the expression levels of these genes were significantly lower in the fruit peel of *MdWRKY31*‐OE tomato fruit than that in the WT at the same stage (Figure S2a). Similarly, we observed a comparable trend in the gel tissue of *MdWRKY31*‐OE tomato fruit (Figure S2b). These findings provide further compelling evidence that MdWRKY31 involves in fruit softening by modulating the expression of cell wall‐modifying enzymes.

### MdWRKY31 transcriptionally represses *MdXTH2* expression

**Figure 5:**
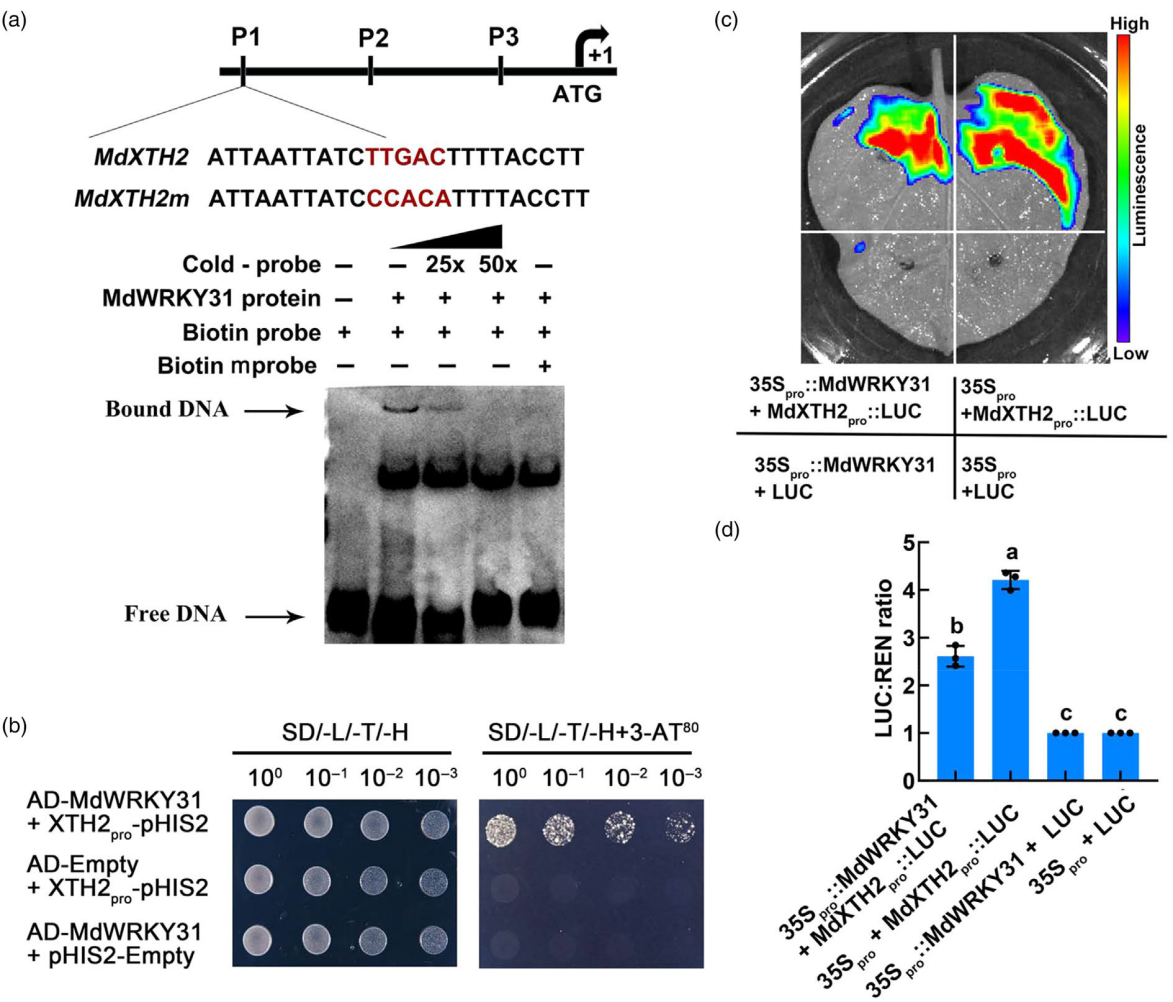
MdWRKY31 negatively regulates *MdXTH2* transcription. (a) Electrophoretic mobility shift assay (EMSA) shows that the MdWRKY31‐GST protein binds directly to the *MdXTH2* promoter. MdXTH2m represents the mutation probe. Lane 1, EMSA with GST protein and biotin probe; Lane 2, EMSA with MdWRKY31 protein and biotin probe; Lane 3, EMSA with MdWRKY31 protein, biotin probe and competitive probe; Lane 4, Increase in competition probe concentration based on lane 3; Lane 5, EMSA with mutation probe (5‐TTGAC‐3 motif was replaced by 5‐CCACA‐3). (b) Yeast one‐hybrid (Y1H) assays showing that MdWRKY31 binds to the promoter fragments of *MdXTH2*. The basal concentration of 3‐AT was 80 mM. AD‐Empty + MdXTH2_pro_‐pHIS2 and AD‐MdWRKY31 + pHIS2‐Empty were used as negative controls. (c) Luciferase assay of transient infection of tobacco leaves showed that MdWRKY31 inhibited *MdXTH2* transcription. Representative images of *Nicotiana benthamiana* leaves 3 days after infiltration. 35S*pro* + LUC as negative control. (d) Relative LUC activity at different injection sites. Luminescence is expressed as a ratio of the LUC to REN signals. Means and standard deviations were calculated from the results of and three technical repetitions. Error bars indicate technical triplicates with error bars reflecting SD. *P* < 0.05 by one‐way ANOVA analysis. Different letters indicate significant difference.

To further elucidate the regulatory role of *MdWRKY31* in controlling fruit firmness, we conducted a comprehensive analysis of the promoters of differentially expressed genes associated with cell wall‐modifying. We found that only the promoters of three cell wall degrading and modifying‐related genes, *MdPG1*, *MdEXPA3*, and *MdXTH2*, contained potential binding motifs for MdWRKY31. Concretely, the promoter of *MdPG1* contains three W‐box *cis*‐acting elements (P1: −1916 bp to −1912 bp, P2: −1233 bp to −1228 bp, and P3: −1053 bp to −1049 bp) (Figure S3), that of *MdEXPA3* contains one W‐box *cis*‐acting element (−617 bp to −612 bp) (Figure S3), and that of *MdXTH2* contains three W‐box *cis*‐acting elements (P1: −1684 bp to −1679 bp, P2: −958 bp to −953 bp, and P3: −16 bp to −11 bp) (Figure 5a). We further performed an electrophoretic mobility shift assay (EMSA) using the MdWRKY31‐GST protein. The results demonstrated that MdWRKY31‐GST exhibited specific binding to the first W‐box *cis*‐acting element in the *MdXTH2* promoter and could not bind specifically to the *MdPG1* and *MdEXPA3* promoters (Figure 5a; Figure S3). The DNA‐MdWRKY31 protein complex bands gradually weakened with increasing concentrations of competing probes, while no such complex bands were observed with the addition of the biotin‐labelled mutant probe (TTGAC mutated to CCACA) (Figure 5a). These findings strongly support the notion that the MdWRKY31‐GST protein specifically binds to the *MdXTH2* promoter via its W‐box recognition sequence. For further validating their binding *in vitro*, we carried out yeast one‐hybrid (Y1H) assays. Co‐expression of MdXTH2pro::pHIS with MdWRKY31‐AD in co‐transformed yeasts enabled their growth on the SD/−Leu/−Trp‐deficient medium, while controls (MdXTH2pro::pHIS+AD; MdWRKY31‐AD+pHIS) failed to grow (Figure 5b). These results confirm the *in vitro* binding of MdWRKY31 to the *MdXTH2* gene promoter.

To investigate the transcriptional regulation of *MdXTH2* by MdWRKY31, we performed dual‐luciferase promoter analysis. For this purpose, we fused the *MdXTH2* 2 kb promoter region to the pGreenII0800‐LUC vector (MdXTH2_pro_::LUC) and the *MdWRKY31* CDS region to the 62SK vector containing the 35S strong promoter (35S_pro_::MdWRKY31). Four vector combinations (35S_pro_::MdWRKY31 + MdXTH2_pro_::LUC, 35S_pro_ + MdXTH2_pro_::LUC, 35S_pro_::MdWRKY31 + LUC, and 35S_pro_ + LUC) were injected into tobacco leaves (Figure 5c). The results demonstrated that compared with the control 35S_pro_ + MdXTH2_pro_::LUC, the co‐expression of 35S_pro_::MdWRKY31 and MdXTH2_pro_::LUC exhibited a weaker relative fluorescence intensity (Figure 5d). Consequently, these results provide robust evidence that MdWRKY31 exerts a negative regulatory effect on the downstream target gene *MdXTH2*.

### *MdWRKY31* negatively regulates fruit softening

**Figure 6:**
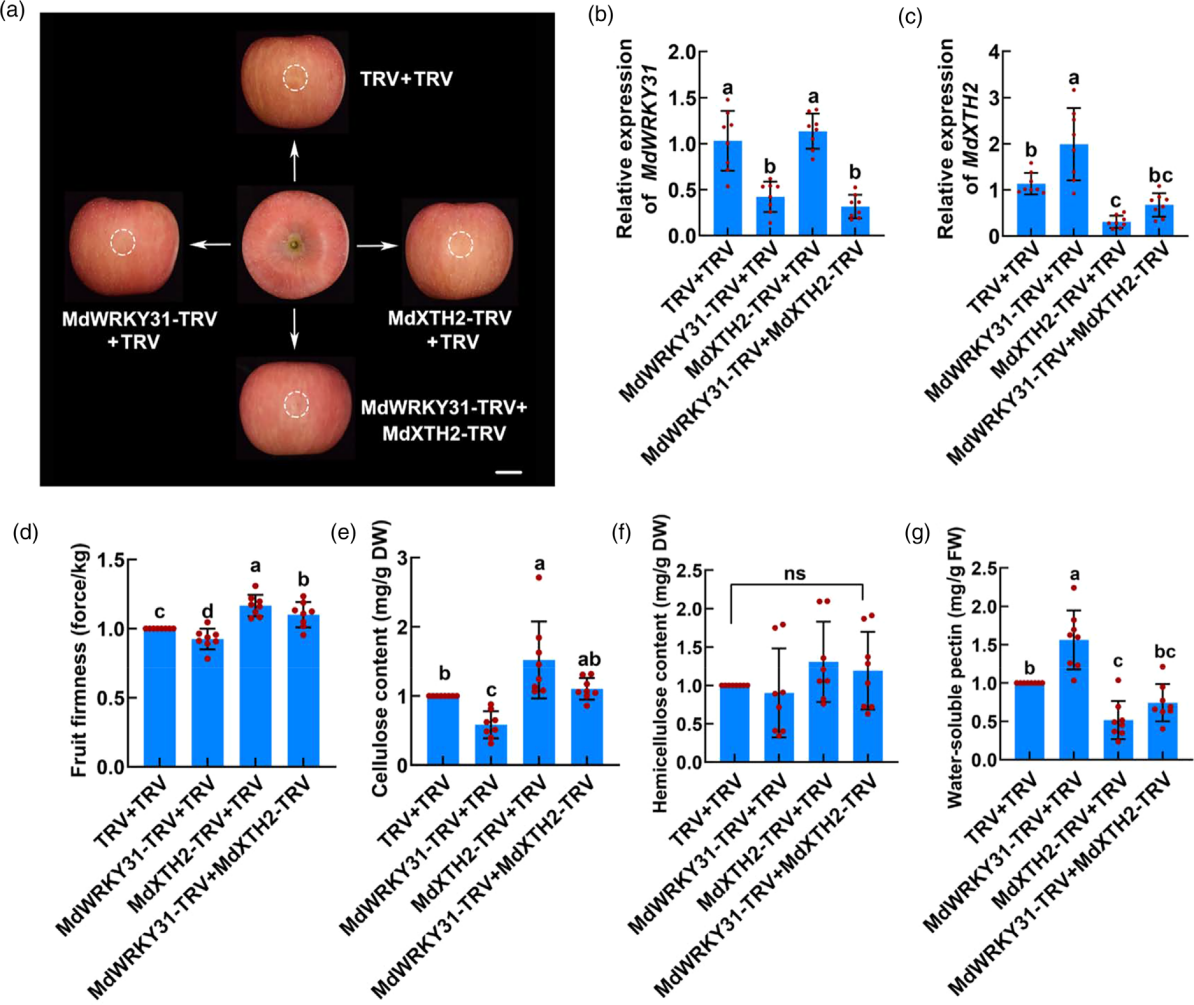
MdWRKY31 negatively modulates fruit softening by regulating *MdXTH2* expression in apple. (a) Transient inhibition of *MdWRKY31* and *MdXTH2* expression by VIGS silencing based on viral vector transformation. TRV empty vector was used as a control. Scale bar = 1 cm. (b, c) Reverse transcription quantitative PCR (RT‐qPCR) analysis of the relative expression levels of *MdWRKY31* (b) and *MdXTH2* (c) genes around the injected sites. (d–g) firmness (d), cellulose (e), hemicellulose (f), and water‐soluble pectin (g) contents in injected apple fruit. Each point in each experiment has eight repetitions. Different letters above groups represent significant differences, shared letters represent no significant differences. For multiple‐group comparison, one‐way ANOVA analysis was performed. Data are shown as the mean ± SD. ns, non‐significant.

To elucidate the role of MdWRKY31 in fruit firmness regulation, we employed the virus‐induced gene silencing (VIGS) system to transiently inject gene silencing‐related vectors (MdWRKY31‐TRV and MdXTH2‐TRV) into apple fruit (Figure 6a). The TRV empty vector was used as a control. RT‐qPCR assays confirmed that MdWRKY31‐TRV and MdXTH2‐TRV successfully functioned as repressors of gene expression (Figure 6b,c). Subsequently, we evaluated the impact of gene silencing on fruit firmness as well as the contents of cellulose, hemicellulose, and soluble pectin. When *MdWRKY31* expression was inhibited compared with that of the control, fruit firmness, and cellulose content were significantly decreased (Figure 6d,e), while hemicellulose content remained relatively unchanged (Figure 6f); moreover, the soluble pectin content increased significantly (Figure 6g). Conversely, injection of the MdXTH2‐TRV vector resulted in increased fruit firmness and cellulose content (Figure 6d,e), minimal changes in hemicellulose content (Figure 6f), and maintenance of lower soluble pectin content (Figure 6g). These findings indicated that both MdWRKY31 and MdXTH2 were involved in regulating fruit softening in apples, with opposite effects: overexpressing *MdWRKY31* increased fruit firmness and inhibited softening, whereas overexpressing *MdXTH2* decreased fruit firmness and promoted softening.

Furthermore, apple fruit that co‐silenced *MdWRKY31* and *MdXTH2* had lower *MdXTH2* expression and soluble pectin content, as well as higher fruit firmness and cellulose content than those that silenced only *MdWRKY31* (Figure 6c–g). However, there were no significant differences in *MdWRKY31* expression, fruit firmness, cellulose content, hemicellulose content, and soluble pectin content in apple fruit co‐injected with MdWRKY31‐TRV and MdXTH2‐TRV as well as MdWRKY31‐TRV alone (Figure 6b,d–g). These results reveal that MdWRKY31 genetically acts upstream of *MdXTH2* and co‐regulates apple fruit softening by transcriptionally repressing the expression of *MdXTH2*.

### Interaction between MdNAC7 and MdWRKY31 hinders the transcriptional repression of *MdXTH2* by MdWRKY31

**Figure 7:**
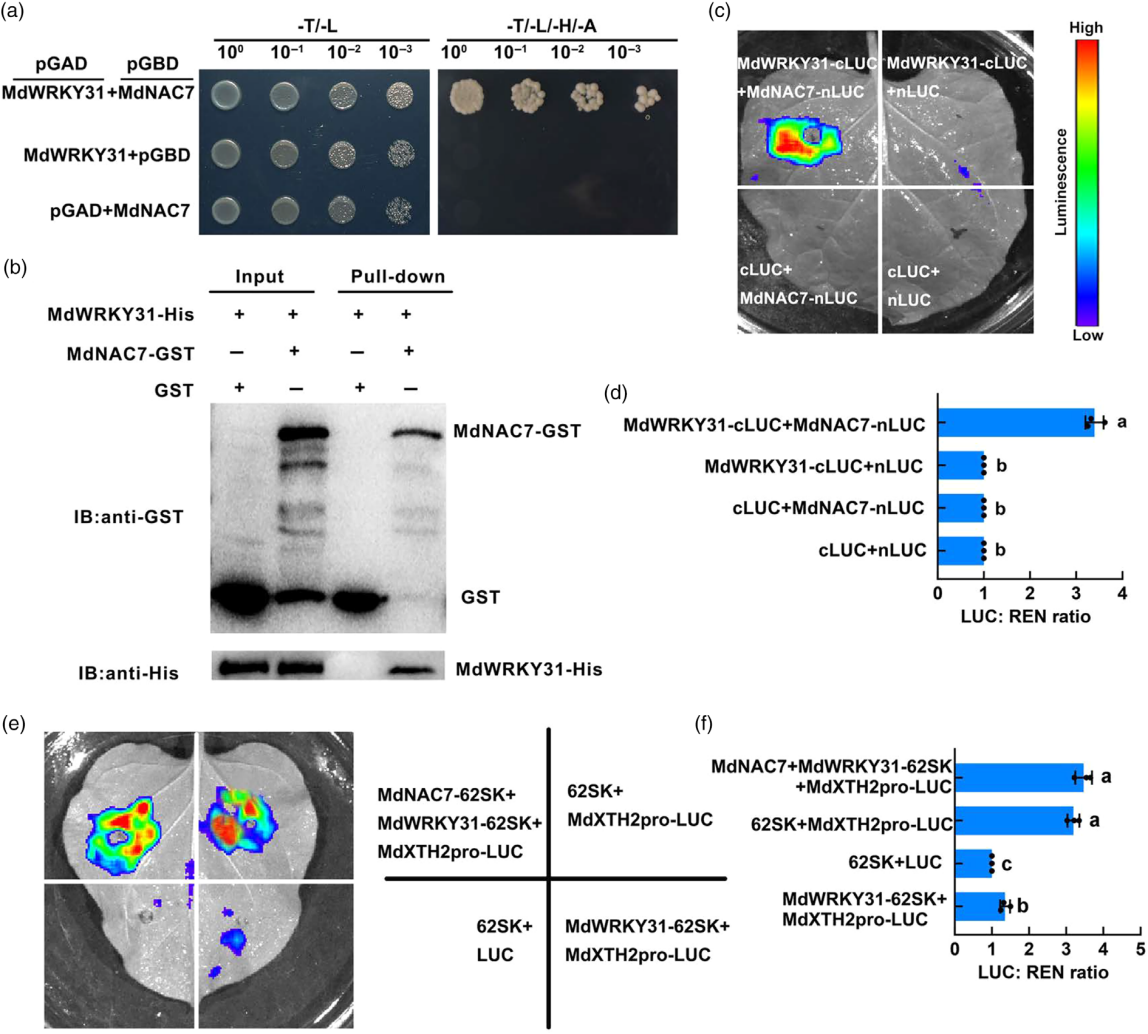
MdNAC7 interacts with MdWRKY31 to alleviate the transcriptional inhibition effect of MdWRKY31 on *MdXTH2*. (a) Yeast two‐hybrid (Y2H) assay to determine the interacting of MdNAC7 and MdWRKY31. pGBD‐MdNAC7/pGAD and pGBD/pGAD‐MdWRKY31 were negative controls. Different co‐transformed AH109 yeast cells were dropped on a culture medium (SD/−Trp/−Leu/−Ade/‐His). (b) *In vitro* GST pull‐down between MdWRKY31 and MdNAC7. The purified MdWRKY31‐His protein was incubated with MdNAC7‐GST and GST control. Proteins were eluted from the magnetic beads and immunoblotted with anti‐His antibody and anti‐GST antibody. (c) MdWRKY31 interacted with MdERF72 in a LUC assay using tobacco leaf cells. MdNAC7‐nLUC and MdWRKY31‐cLUC interacted in the nucleus of tobacco leaf cells. (d) The interact ability was expressed as a ratio of LUC to REN. Data values represent three independent experiments with similar results, and SD in all the points is <5%. (e) MdWRKY31‐MdNAC7 interaction alleviates the transcriptional repression of *MdXTH2* by MdWRKY31. (f) Quantitative statistics of fluorescence intensity. Data are averages ± SD from three independent experiments.

To gain deeper insights into the regulation of MdWRKY31 in fruit softening, we conducted a yeast two‐hybrid (Y2H) screen assay to identify potential interacting proteins of MdWRKY31. The results of the Y2H screen identified a NAC family TF called MdNAC7. To validate this interaction, we performed point‐to‐point validation experiments using a yeast two‐hybrid system. The full‐length cDNA of *MdWRKY31* was fused to the DNA‐binding domain (BD) in the pGBT9 vector while that of *MdNAC7* was fused to the activation domain (AD) in the pGAD424 vector. Yeast cells co‐expressing MdWRKY31‐BD and MdNAC7‐AD were able to grow on a selective medium (yeast four‐deficiency medium SD/−L/−T/−H/−A), confirming protein–protein interactions (Figure 7a). Pull‐down results showed that MdNAC7‐GST enriched the His‐MdWRKY31 protein, while the GST control did not show such enrichment (Figure 7b), which further confirms the interaction between MdWRKY31 and MdNAC7 *in vitro*. *In vivo* validation of the MdWRKY31–MdNAC7 interaction was performed using transient luciferase reporter assays in leaves of *Nicotiana benthamiana* (*N. benthamiana*) (Figure 7c). Co‐expression of MdWRKY31‐cLUC and MdNAC7‐nLUC reconstituted a functional LUC vector in the nucleus, whereas control combinations (MdWRKY31‐cLUC+nLUC, cLUC+MdNAC7‐nLUC, and cLUC+nLUC) did not show significant fluorescence intensity (Figure 7d). These results solidify the notion that MdWRKY31 physically interacts with MdNAC7 in plants.

To assess whether the MdWRKY31–MdNAC7 interaction impacts the transcriptional repression of the downstream target gene *MdXTH2* by MdWRKY31, we employed a dual‐luciferase reporter gene assay (Figure 7e). Co‐injection of 35S_pro_::MdWRKY31 and MdXTH2_pro_::LUC resulted in reduced LUC activity, but it was restored upon co‐injection of 35S_pro_::MdNAC7 (Figure 7f). This result indicates that the interaction between MdWRKY31 and MdNAC7 suppresses the effect of MdWRKY31 on *MdXTH2* expression.

**Figure 8:**
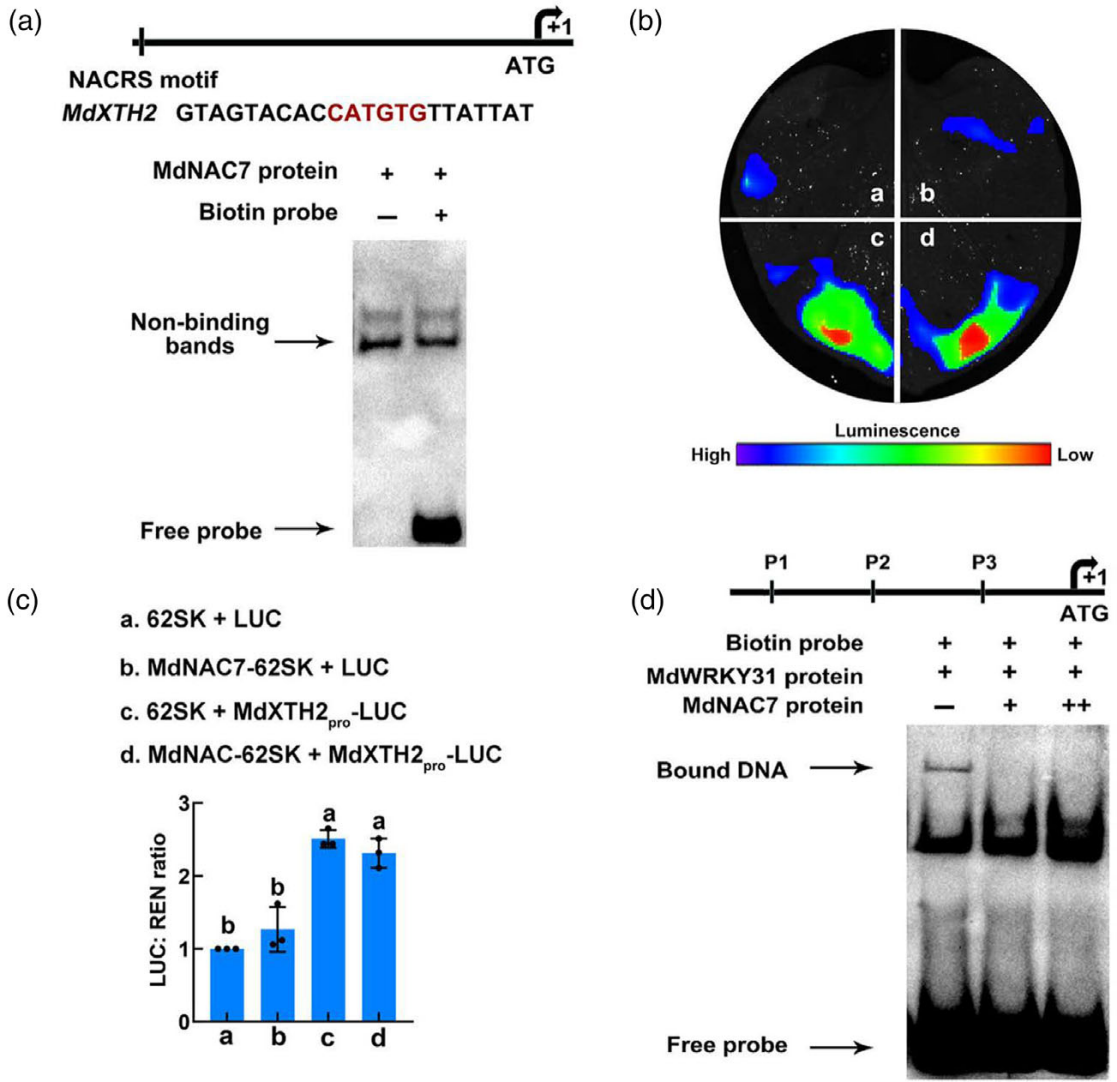
MdNAC7 does not directly regulates *MdXTH2* expression, but the protein complex formed with MdWRKY31 hinders MdWRKY31 from binding to the promoter of *MdXTH2*. (a) EMSA experiments show that the MdNAC7 protein not binds to the *MdXTH2* promoter. Lane 1, EMSA with MdNAC7 protein; Lane 2, EMSA with MdNAC7 protein and biotin probe. (b) Relative LUC activity at different injection sites. Representative images of *N. benthamiana* leaves 3 days after infiltration. (c) Quantitative statistics of fluorescence intensity. 62SK + LUC as negative control. Means and standard deviations were calculated from the results of and three technical repetitions. Error bars indicate technical triplicates with error bars reflecting SD. *P* < 0.05 by one‐way ANOVA analysis. Different letters indicate significant difference. (d) EMSA experiments show that interaction of MdNAC7 with MdWRKY31 hinders MdWRKY31 from binding to the *MdXTH2* promoter. In the context of co‐incubation of MdWRKY31 protein with *MdXTH2* biotin probe, MdNAC7 protein was added and the binding bands disappeared.

Subsequently, we analysed the *cis*‐acting elements of NAC TFs on the *MdXTH2* promoter to explore whether MdNAC7 has a potential regulatory effect on *MdXTH2*. As shown in Figure S4, there is a NACRS *cis*‐acting element on the *MdXTH2* promoter. However, no binding bands were detected in the EMSA assay (Figure 8a). Meanwhile, a subsequent double luciferase assay revealed that the fluorescence intensity of MdNAC7‐62SK co‐injected with MdXTH2_pro_‐LUC was not significantly different than that of the control (62SK + MdXTH2_pro_‐LUC) (Figure 8b,c). These results suggest that MdNAC7 does not directly bind *MdXTH2* promoter and regulate its expression. Thus, we concluded that the return of LUC fluorescence intensity to the control level after co‐injection of MdNAC7‐62SK and MdWRKY31‐62SK with MdXTH2_pro_‐LUC into *N. benthamiana* leaves is due to forming a protein complex MdWRKY31–MdNAC7 that inhibits the binding of MdWRKY31 to the *MdXTH2* promoter (Figure 7e,f).

To determine whether MdWRKY31, which exists as the MdWRKY31‐MdNAC7 protein complex, binds to the W‐box *cis*‐acting element of *MdXTH2* promoter, we performed an EMSA study. As shown in Figure 8d, MdNAC7 competed with the W‐box *cis*‐acting element of *MdXTH2* promoter for binding to the MdWRKY31 protein, as evident from the disappearance of the binding band of MdWRKY31 to the W‐box *cis*‐acting element of *MdXTH2* promoter upon the co‐addition of MdWRKY31 and MdNAC7 proteins. This result suggests that MdWRKY31 cannot bind the *MdXTH2* promoter when WRKY31 is present as the protein complex MdWRKY31‐MdNAC7, which well explains that the interaction between MdWRKY31 and MdNAC7 hinders the transcriptional repression of *MdXTH2* by MdWRKY31.

### MdNAC7 positively regulates apple fruit softening

**Figure 9:**
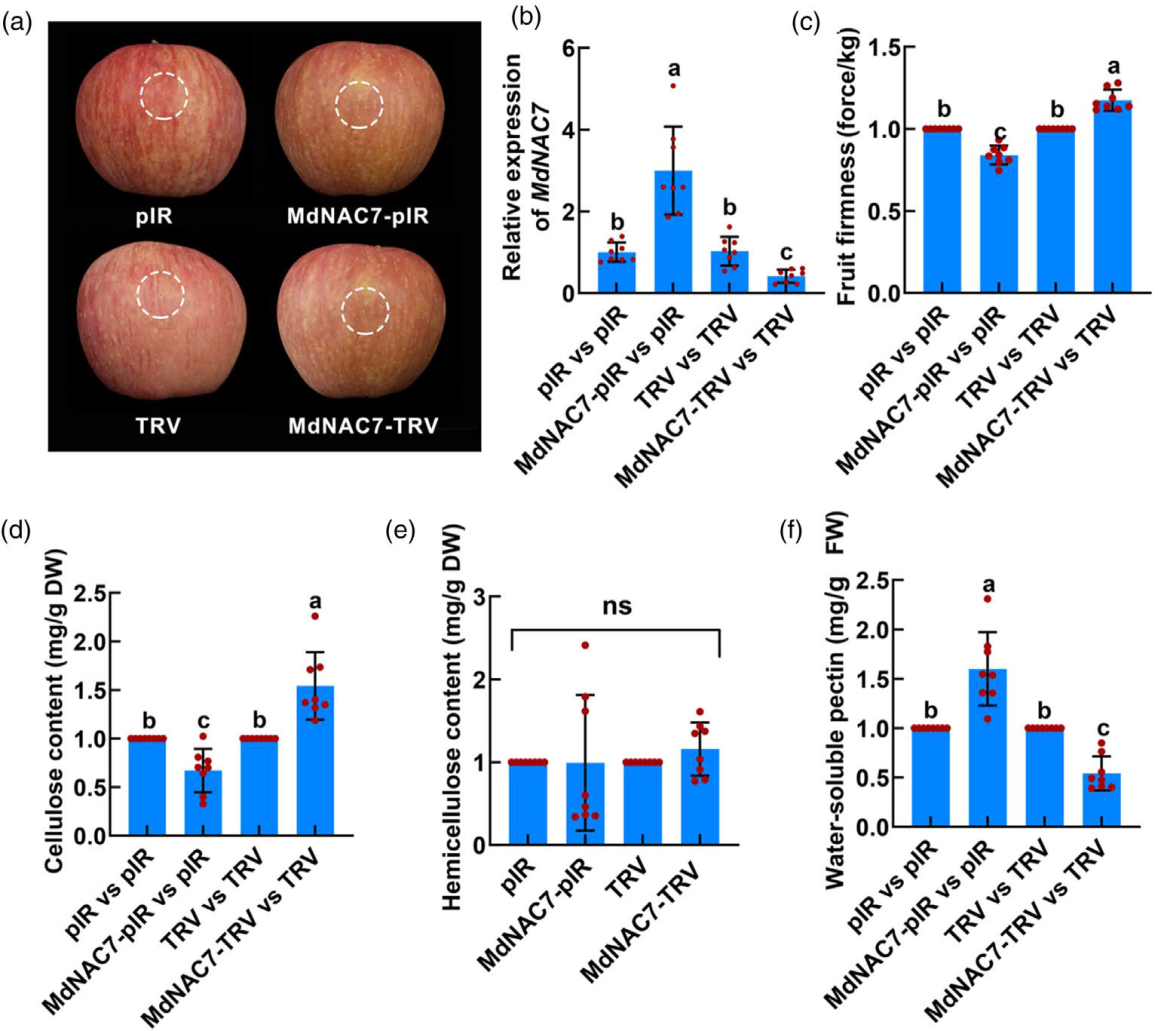
MdNAC7 positively regulates fruit softening in apple. (a) Transient inhibition of *MdNAC7* and overexpression of *MdNAC7* by viral vector‐based transformation. TRV and pIR empty vectors were used as controls. Scale bar = 1 cm. (b) RT‐qPCR analysis of the relative expression levels of *MdNAC7* gene around the injected sites. (c–f) Hardness (c), cellulose (d), hemicellulose (e), and water‐soluble pectin (f) contents in injected apple fruit. (b–f) Four replicates for each group and the experiment were repeated at least eight times. Differences between groups were analysed by one‐way analysis of variance (ANOVA). Data represents the mean ± SD. Histograms in selected figures labelled with different letters represent significant differences.

We have demonstrated that MdNAC7 is involved in fruit softening by interacting with MdWRKY31 and relieving the transcriptional repression of *MdXTH2* by MdWRKY31 (Figures 7 and 8). To further clear the role of MdNAC7 in regulating fruit softening, we performed viral transient silencing and overexpression vectors (MdNAC7‐TRV and MdNAC7‐pIR, respectively) to manipulate the expression of *MdNAC7* in apple fruit (Figure 9a). TRV and pIR were used as negative controls for the empty vectors, respectively. MdNAC7‐TRV injection significantly downregulated *MdNAC7* expression in apple fruit, implying that MdNAC7‐TRV effectively silenced *MdNAC7* expression (Figure 9b), leading to increased fruit firmness and cellulose content (Figure 9c,d), relatively unchanged hemicellulose content (Figure 9e), as well as significantly reduced soluble pectin content (Figure 9f) compared with that in the negative control. Conversely, apple fruit injected with MdNAC7‐pIR showed significantly higher *MdNAC7* expression, which was threefold higher compared with that of the empty vector control (Figure 9b). We further observed that fruit firmness and cellulose content were significantly decreased (Figure 9c,d), hemicellulose remained almost unchanged (Figure 9e), as well as soluble pectin content was significantly increased (Figure 9f). These results indicate that MdNAC7 positively regulates fruit softening in apple.

### MdNAC7 functions as a positive response factor to ethylene signalling

**Figure 10:**
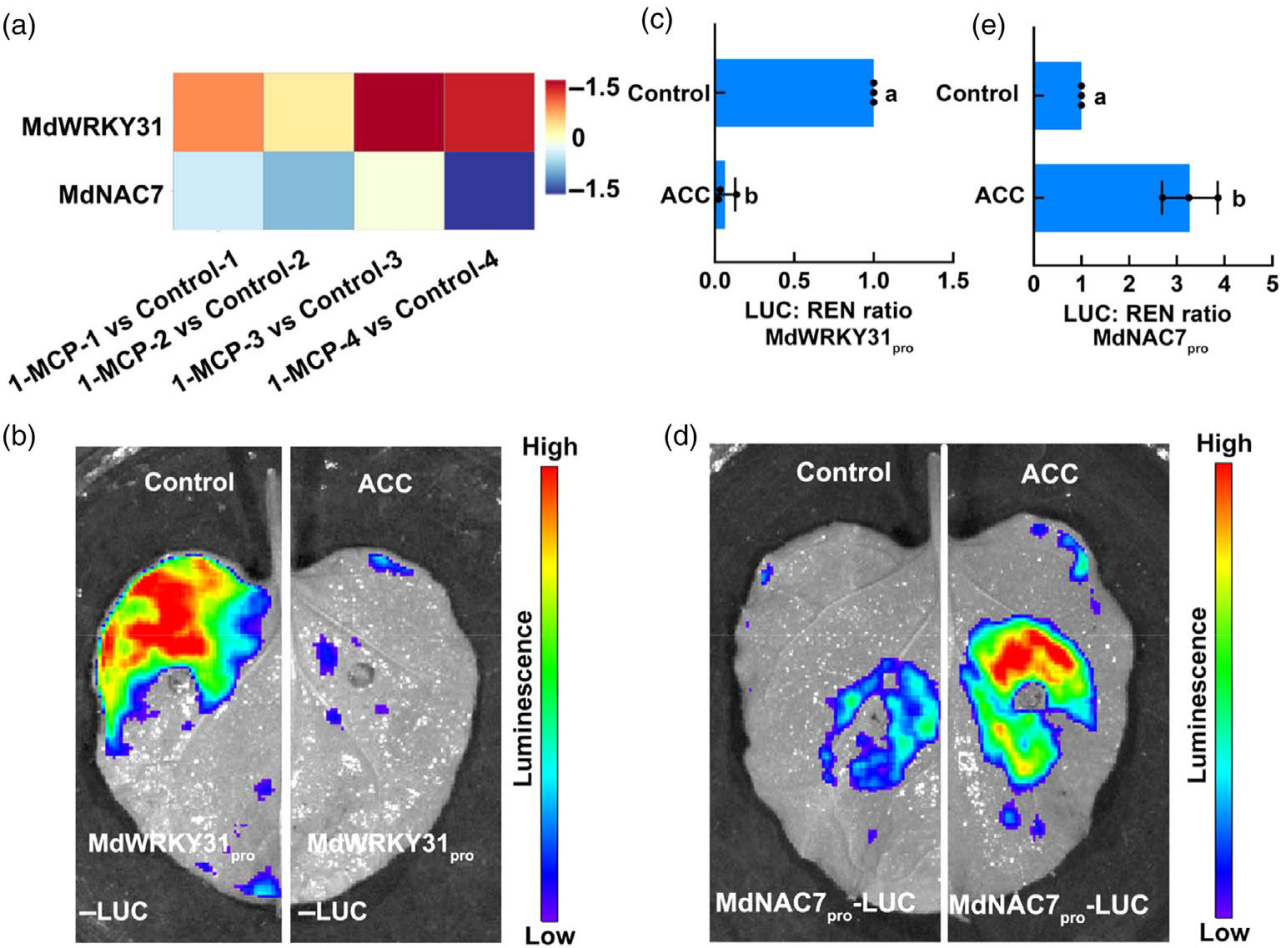
Ethylene represses and induces the expression of *MdWRKY31* and *MdNAC7*, respectively. (a) Relative gene expression of *MdNAC7* and *MdWRKY31* in 1‐MCP treatment transcriptome data. (b) Transient expression assay showing that ethylene represses *MdWRKY31* expression. (c) Relative LUC activity of *MdWRKY31* promoter at different injection sites. (d) Transient expression assay showing that ethylene induces *MdNAC7* expression. (e) Relative LUC activity of *MdNAC7* promoter at different injection sites. 20 μM 1‐aminocyclopropane‐1‐carboxylic acid (ACC) treated tobacco leaves after injection and water was used as a control. Three technical replications were performed for each experiment. Different letters indicate significant differences (*t*‐test, *P* < 0.05). Data represents the mean ± SD.

In this study, we found that MdNAC7 promotes fruit softening by interacting with MdWRKY31 and relieving the transcriptional repression of *MdXTH2* by MdWRKY31. In our previous study, we discovered that ethylene has a negative impact on the expression of *MdWRKY31* (Wang *et al*., 2023b). Therefore, we further aimed to verify whether *MdNAC7* responds to ethylene in order to regulate fruit firmness through the formation of the MdNAC7–MdWRKY31–MdXTH2 regulatory network. By analysing the transcriptome of apple fruit treated with 1‐MCP, we observed a significant upregulation of *MdWRKY31* expression and a significant downregulation of *MdNAC7* expression after 1‐MCP treatment (Figure 10a). This suggests that *MdNAC7* may be positively regulated by ethylene signalling. We further conducted LUC experiments to observe the response of *MdNAC7* to ethylene. MdWRKY31_pro_‐LUC and MdNAC7_pro_‐LUC vectors were constructed and injected into *N. benthamiana* leaves, which were subsequently treated with 1‐aminocyclopropanecarboxylic acid (ACC), while solvent water was used as a control (Figure 10b,d). The LUC fluorescence intensity was measured, and the results revealed that after ACC treatment, the fluorescence intensity of tobacco leaves injected with MdWRKY31_pro_‐LUC decreased compared with that of the control (Figure 10c), consistent with our prior findings and demonstrating the feasibility of verifying gene response to ethylene using this approach. Furthermore, this experiment resulted in an enhancement in fluorescence intensity, further confirming that *MdNAC7* is positively regulated by ethylene (Figure 10e). Based on these findings, we propose that the regulatory complex MdNAC7–MdWRKY31–MdXTH2 bridges ethylene signalling and fruit softening, and finally analysed the molecular mechanism of ethylene‐mediated apple fruit softening.

## Discussion

It has long been recognized that reduction in fruit firmness is closely associated with an increase in ethylene production during fruit ripening (Bu *et al*., 2013; Harb *et al*., 2012; Tatsuki *et al*., 2013). Especially, during the softening of climacteric fruit, the degradation of pectin and cellulose is dependent on the presence of ethylene (Ergun *et al*., 2005; Nishiyama *et al*., 2007), but the molecular mechanism has not been identified. Here, we demonstrate that MdWRKY31 interacts with MdNAC7 in response to ethylene, transcriptionally repressing MdXTH2, a xyloglucan endotransglucosylase/hydrolase (XTH) which plays a vital role in fruit softening, thus reducing apple fruit firmness. By uncovering the regulatory mechanisms underlying fruit softening, we hope to contribute to the development of strategies to extend shelf life and maintain fruit quality.

Ethylene is a key regulator of fruit ripening and plays a crucial role in inducing fruit softening by influencing the expression of genes involved in cell wall modification (Felten *et al*., 2018; Johnston *et al*., 2009; Wang *et al*., 2009). Inhibition of polygalacturonase 1 (*PG1*) expression makes apples more resistant to storage and less susceptible to softening (Atkinson *et al*., 2002, 2012; Tacken *et al*., 2010). In addition to PG1, the gene encoding β‐gal is also regulated by ethylene and contributes to fruit softening in apple, tomato, and avocado (Moctezuma *et al*., 2003; Smith *et al*., 2002; Tateishi *et al*., 2007; Wei *et al*., 2012). Interestingly, in tomatoes, a specific gene from the ERF.F subfamily, known as SlERF.F12, acts as a suppressor of fruit ripening and softening. It achieves this by recruiting the co‐repressor TPL2 and histone deacetylases HDA1/HDA3 to epigenetically suppress the expression of ripening‐related genes, including *ACS2*, *ACS4*, *PG2a*, and pectin lyase (Deng *et al*., 2022).

Here, we show that *MdXTH2* is positively regulated by ethylene. XTH is considered crucial for fruit softening, as it plays a pivotal role in breaking down xyloglucan and loosening the cell wall to facilitate further modification by other cell wall‐related enzymes (Atkinson *et al*., 2009). In ripe apple fruit, all *MdXTHs* are expressed, with *MdXTH2*, *MdXTH10*, and *MdXTH11* showing the highest expression levels (Muñoz‐Bertomeu *et al*., 2013). Furthermore, silencing of *MdXTH2* expression improves apple fruit firmness (Figure 6). Our previous study also found that in transgenic tomatoes overexpressing both *MdXTH2* and *MdXTH10*, the expression levels of fruit softening‐related genes (*SlPG2A*, *SlXTHs*, *SlCel2*, and *SlTBG4*), as well as genes involved in ethylene biosynthesis and signalling pathways (*SlACS2*, *SlACO1*, and *SlERF2*), were significantly upregulated (Zhang *et al*., 2017). Similarly, in the current study, RT‐qPCR analysis also showed that the expression levels of cell wall‐modifying genes (*MdEXPA3*, *MdPL1*, *MdXTH10*, and *MdEG1*) as well as ethylene biosynthesis‐related genes (*MdACS1* and *MdACO1*) were correspondingly downregulated following the silencing of *MdXTH2* (Figure S5). Overall, our findings not only further confirmed that ethylene plays an essential role in apple fruit softening through its ability to regulate several cell wall hydrolysis‐related genes, but also approved that *MdXTH2* acts as the key cell wall‐modifying gene to dominate ethylene‐induced fruit softening in apple.

As evident in Figures 5, 6, MdWRKY31 plays a crucial role in regulating MdXTH2‐modulated fruit softening and also is negatively regulated by ethylene signalling (Figure 10a–c). Transcriptome sequencing analysis of MdWRKY31‐OVX and WT apple plants revealed that in MdWRKY31‐OVX plants, most genes involved in cell wall‐modifying exhibited significant downregulation compared with that in WT plants (Figure 4). This suggests that other WRKY transcription factors in various species may similarly regulate fruit softening by modulating the expression of genes associated with cell wall modification. For instance, in *Fragaria vesca*, FvWRKY48 binds to the PL *FvPLA* promoter, thereby controlling fruit softening (Zhang *et al*., 2022). Therefore, we speculate that the molecular mechanism through which WRKY TFs regulate fruit firmness by regulating the expression of cell wall modification‐associated genes could be conserved across different species. What is more, apart from *MdXTH2*, which is directly regulated by MdWRKY31 at the transcriptional level, the expression of other cell wall modification‐related genes may be indirectly influenced by MdWRKY31. Notably, PL, a gene known to be the major contributor to softening (Uluisik *et al*., 2016), is negatively regulated by MdWRKY31. However, it is not directly transcriptionally regulated by MdWRKY31 as a direct downstream target gene (Figure 4c,d). Further research is required to elucidate the precise mechanisms by which MdWRKY31 indirectly regulates each of these genes.

Interestingly, we also found that *MdWRKY31*‐overexpressing tomato fruit was significantly smaller than WT during fruit ripening (Figure 2a; Figure S6). Previous studies have shown that WRKYs play a key role in regulating fruit softening and fruit development, especially fruit size (Chen *et al*., 2023; Rehman *et al*., 2024). For example, suppression of *Sl‐AGL11* (*TAGL11*), a class D gene, results in smaller and lighter seeds, while up‐regulating *Sl‐AGL11* leads to fleshy sepals, a hypertrophic placenta, and extreme softening before initiation of ripening (Huang *et al*., 2017; Ocarez and Mejía, 2016). Inhibition of *SlMBP3* resulted in a similar inhibition of chamber liquefaction as reported here but likewise produced other phenotypes, including altered seed coat development, reduced seed viability, and significantly reduced fruit size (Huang *et al*., 2021; Kim *et al*., 2022). The transcriptional enrichment of expansion protein *SlExp1* and endoglucanase *SlCel2* affects not only fruit softening but also fruit size (Su *et al*., 2024). *SlLOB1*‐repressed fruit showed a smaller size than WT during fruit ripening (Shi *et al*., 2021). In this study, we found that MdWRKY31 forms a transcriptional complex with MdNAC7 to regulate fruit firmness. Previous studies have shown that multiple NACs directly regulate tomato fruit development and size (Gao *et al*., 2018; Peng *et al*., 2023; Zhang *et al*., 2021). Based on the above clues, we speculate that the main reason why *MdWRKY31*‐overexpressing tomato fruit are significantly smaller than WT may be the following three points: firstly, MdWRKY31 directly regulates the expression of fruit development‐related genes; secondly, MdWRKY31 regulates fruit size by modulating cell wall degradation and modification; thirdly, MdWRKY31 indirectly regulates fruit size by forming a complex with MdNAC7. These speculations need to be further confirmed in the future.

Additionally, in the current study, we also demonstrated the critical role of the ethylene‐responsive NAC family TF MdNAC7 in fruit softening. Through interactions with MdWRKY31, MdNAC7 acts to suppress transcriptional repression of downstream target genes, ultimately resulting in reduced fruit firmness and enhanced fruit softening. The NAC family is one of the largest families of plant‐specific TFs. For instance, NOR is a well‐studied member of the NAC family, with the non‐mature mutant (*nor*) being a natural mutant in tomatoes with an evident inhibition of maturation phenotype (Barry and Giovannoni, 2007). Another member, FaRIF, from the NAC (NAM, ATAF, and CUC) family has been shown to be involved in cell wall reorganization during strawberry fruit ripening, with FaRIF‐RNAi mutants displaying increased fruit firmness (Martin‐Pizarro *et al*., 2021). Our experiment findings indicate that MdNAC7 promotes fruit softening when transiently injected in apple fruit (Figure 9). At the late stage of fruit ripening, the gradual increase in ethylene content stimulates *MdNAC7* expression (Figure 10a,d,e). Subsequently, MdNAC7 interacts with MdWRKY31, effectively relieving the transcriptional repression of *MdXTH2* that is imposed by MdWRKY31 (Figure 7). This interaction leads to the increased expression of *MdXTH2*, ultimately contributing to fruit softening. To determine whether MdWRKY31 binds to the W‐box *cis*‐acting element of the *MdXTH2* promoter as a MdWRKY31‐MdNAC7 protein complex or as an individual protein monomer, we performed an EMSA study. The results confirmed that MdNAC7 competes with the *MdXTH2* promoter for binding to the MdWRKY31 protein, as evident from the disappearance of the binding band of MdWRKY31 to the *MdXTH2* promoter upon the co‐addition of MdWRKY31 and MdNAC7 proteins (Figure 8d). This result indicates that MdNAC7 and MdWRKY31 interact independently with the *MdXTH2* promoter, rather than forming a complex.

**Figure 11:**
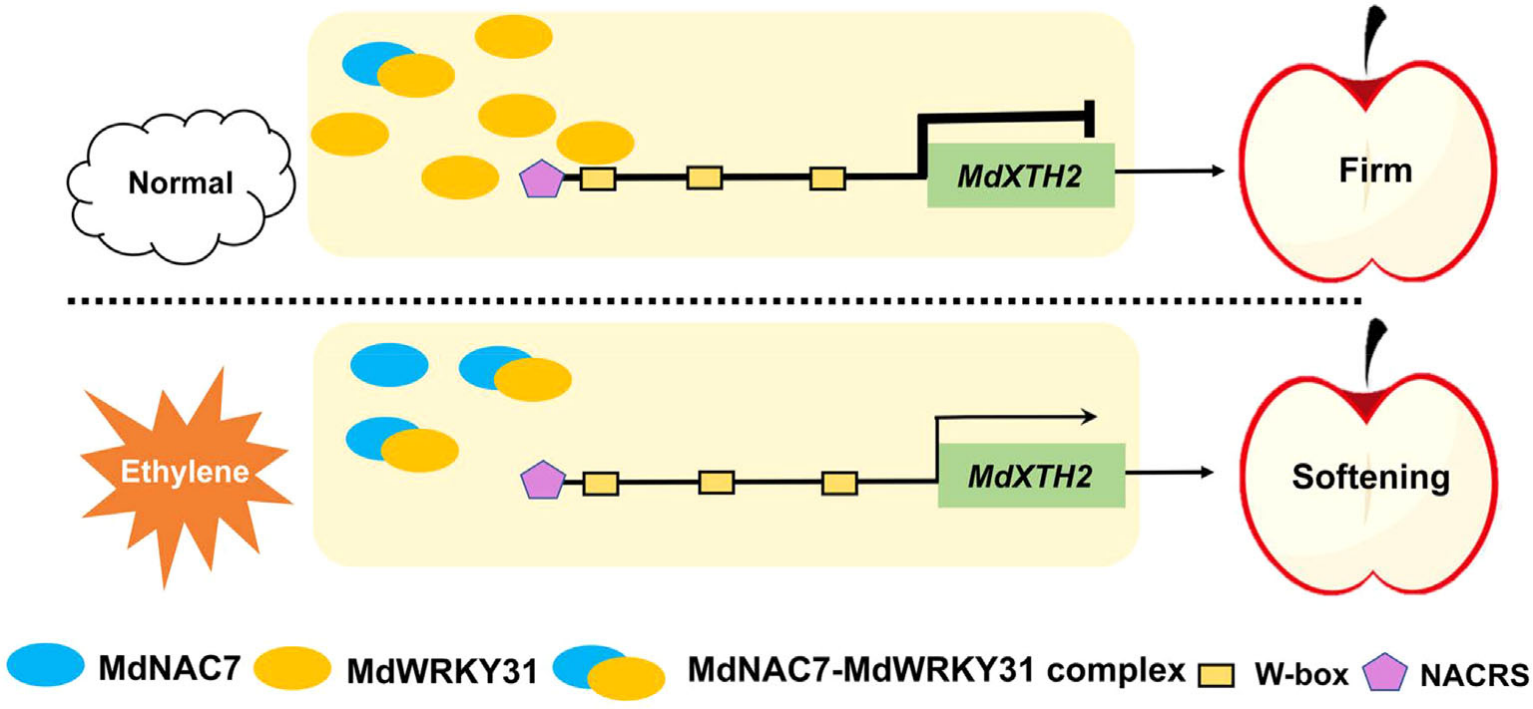
Model showing the regulatory mechanism of MdNAC7‐MdWRKY31 module in regulating apple fruit softening by modulating the expression of *MdXTH2*. Under air conditions, a sufficient amount of the MdWRKY31 protein directly binds to the promoter of *MdXTH2* and represses *MdXTH2* expression, thus delaying fruit softening and maintaining fruit firmness. However, when ethylene is present, ethylene signalling significantly reduces and increases the abundance of MdWRKY31 and MdNAC7, respectively. In this case, almost all MdWRKY31 proteins are interacted by MdNAC7 proteins to form MdWRKY31‐MdNAC7 protein complexes, thereby hindering MdWRKY31 from binding to *MdXTH2* promoter. These occurrences ultimately result in ethylene‐mediated apple fruit softening.

Based on our findings, we propose a model for the regulatory mechanism of MdWRKY31–MdNAC7 in regulating the ethylene‐induced fruit softening in apple (Figure 11). Under air conditions, a sufficient amount of the MdWRKY31 protein directly binds to the promoter of *MdXTH2* and represses *MdXTH2* expression, thus delaying fruit softening and maintaining fruit firmness. However, when ethylene is present, ethylene signalling significantly reduces and increases the abundance of MdWRKY31 and MdNAC7, respectively. In this case, almost all MdWRKY31 proteins are interacted by MdNAC7 proteins to form MdWRKY31‐MdNAC7 protein complexes, thereby hindering MdWRKY31 from binding to *MdXTH2* promoter. These occurrences ultimately result in ethylene‐mediated apple fruit softening. Our research sheds light on the intricate signalling pathways involved in ethylene‐responsive fruit softening and provides a promising avenue for improving postharvest fruit quality through targeted manipulation of *MdWRKY31*. This sheds light on the intricate mechanisms governing fruit firmness and opens avenues for enhancing fruit quality and reducing economic losses associated with softening in apples and other fruit.

## Materials and methods

### Plant materials and growth conditions

The fifth functional leaves of *MdWRKY31*‐overexpressing apple plantlets (MdWRKY31‐OVX5, MdWRKY31‐OVX9, and MdWRKY31‐OVX10) and WT control (*Malus* × *domestica* cv Royal Gala) were selected to detect cellulose, hemicellulose, and soluble pectin contents as well as to measure gene expression.

The classic cultivated tomato (*Solanum lycopersicum*) variety Ailsa Craig (AC; LA2838A) from TGRC (Tomato Genetic Resource Center; https://tgrc.ucdavis.edu) was used as the wild‐type (WT) for this study. Tomato seeds were germinated on moist filter paper at room temperature and then sown in a substrate culture bowl. Seedlings were grown in a growth chamber at 26 °C for 16 h of light (200 μmol/m^2^/s) and 18 °C for 8 h of darkness at a relative humidity of 60%. 30‐day‐old seedlings were transplanted to a greenhouse at the Horticultural Experiment Station of Shandong Agricultural University. The flowering stage was marked and the number of days from flowering to Br stage was recorded for each genotype. Fruit was harvested at 39, 42, 47, and 52 DAF (days after flowering), which corresponded to MG (mature green) stage, BR (breaker stage) stage, B5 (breaker+5d) stage, and B10 (breaker+10d) stage of WT, respectively.

### RNA extraction, reverse transcription PCR, and RT‐qPCR

Total RNA was extracted using an OminiPlant RNA Kit (CWBIO, Beijing, China), following the manufacturer's protocol. Detection of RNA concentration using Nanodrop 2000 spectrophotometer. Reverse transcription was performed using MonScript™ RTIII All‐in‐One Mix with dsDNase (Monad, Wuhan, China). RT‐PCR and RT‐qPCR assays were conducted as per Hu *et al*. (2019). The primers listed in Table S1. The experiment was performed using at least three biological replicates.

### RNA‐Seq analysis

Total RNAs were extracted from apple fifth functional leaves of WT or *MdWRKY31* transgenic fruit trees. RNA‐Seq assays were performed with the methods as described by Hu *et al*. (2019).

### Yeast one‐hybrid (Y1H) and yeast two‐hybrid (Y2H) assays

Y1H experiments were conducted using the Matchmaker Gold Y1H library Screening System (Clontech, Palo Alto, CA). The promoter fragment 2 kb upstream of *MdXTH2* was cloned into the pHIS2 vector (Clontech). The full length of *MdWRKY31* was ligated into the pGAD424 vector (Clontech). The primers used for cloning are listed in Table S1. The cerevisiae strains were then allowed to grow for 3–4 days at 28 °C to assess DNA–protein interactions. And 3‐aminotriazole (3‐AT; Sigma Aldrich) was used for screening. The Y1H assays were performed as previously described (Ren *et al*., 2021).

Y2H screening assay was performed with the methods as described by Wang *et al*. (2023b). Y2H assays were performed using the Matchmaker™ GoldYeast Two‐Hybrid System (Clontech). The coding sequences (CDSs) of *MdNAC7* and *MdWRKY31* were subcloned into pGAD424 and pGBT9 vectors, respectively, and fused with the AD and BD. The pGAD424‐MdNAC7 and pGBT9‐MdWRKY31 plasmids were transformed into yeast strain AH109 using the lithium acetate method. Yeast cells were cultured on a micro‐medium according to the manufacturer's instructions.

### Electrophoretic mobility shift assay (EMSA)

EMSA was performed using the LightShift Chemiluminescent RNA EMSA Kit (Thermo Fisher Scientific, Waltham, MA, USA) as per instructions. Plasmid construction for the expression of recombinant MdWRKY31 protein in *Escherichia coli* (primers are listed in Table S1) and protein purification was conducted as described (Li *et al*., 2013). A standard binding reaction was performed in a total volume of 10 μL by incubating an appropriate amount of purified MdWRKY31 protein with biotin‐labelled probe DNA at room temperature for 30 min. The binding reaction products were resolved on a 6% polyacrylamide gel run in 0.5× Tris–borate–EDTA and transferred onto a nylon membrane for streptavidin–horseradish peroxidase–based detection of bands.

### Transient dual‐luciferase assays and bimolecular fluorescence complementation assay

The 2000 bp promoter regions of *MdXTH2* were cloned into the pGreenII 0800‐LUC double‐reporter vector, while the full CDS was cloned into the pGreenll 62‐SK vector as an effector. The effector plasmid was transformed into tobacco (*Nicotiana benthamiana*) leaves together with the reporter plasmid at a 1:1 ratio. Tobacco plants were cultured at 25 °C for 2–3 days. In the control group, an empty pGreenII 62‐SK vector replaced the effector plasmid at the same concentration. The ratio of firefly (*Photinus pyralis*) relative to REN (*Renilla reniformis*) LUC activity was used as an indicator of the transcriptional efficiency of MdWRKY31 for *MdXTH2* promoter. Primers for vector construct are listed in Table S1.

The full‐length *MdWRKY31* and *MdXTH2* CDSs were inserted into the cLUC and nLUC vectors. *Agrobacterium tumefaciens* (*A. tumefaciens*) GV3101 cells were transformed with the resulting plasmids according to a freeze–thaw method. Equal volumes of the different combinations were mixed for the infiltration of *Nicotiana benthamiana* (*N. benthamiana*) leaves using a needle‐free syringe. Leaves were co‐infiltrated with MdWRKY31‐nLUC + cLUC, nLUC + MdNAC7‐cLUC, and nLUC + cLUC as the negative control. The results are expressed as relative luciferase activity (Firefly LUC/Renilla LUC).

### Pull‐down assay

GST fusion proteins were used for protein interaction analysis. In the GST pull‐down assay, the full‐length cDNA of *MdNAC7* was inserted into the pGEX‐4 T‐1 vector and the full‐length cDNA of *MdWRKY31* was inserted into the pET‐32a vector. Recombinant proteins were used to perform the GST pull‐down assay described by Oh *et al*. (2012). The interaction of MdNAC7 with MdWRKY31 was analysed by SDS‐PAGE and Western blotting.

### Viral vector construction and transient expression in apple fruit

Using apple fruit cDNA as a template, PCR amplified 200–300 bp fragments of the non‐conserved regions of *MdNAC7*, *MdXTH2*, and *MdWRKY31* CDSs to construct antisense expression viral vectors. The PCR products were cloned into the TRV vector under the control of the dual 35S promoter. The gene silencing vectors were named MdNAC7‐TRV, MdXTH2‐TRV, and MdWRKY31‐TRV, respectively, and were introduced into *A. tumefaciens* GV3101. The full‐length cDNA of *MdNAC7* was ligated to the pIR vector to construct an overexpression viral vector named MdNAC7‐pIR.

‘Starking Delicious’ apples are harvested at 130 days after flowering, without mechanical damage, uniform colouring and in a basically uniform state of growth. The recombinant vector was injected into ‘Starking Delicious’ apples using a needleless syringe. The infiltrated fruit was kept in a growth chamber at 25 °C for 5–7 days, and then fruit samples were taken around the injection sites, and frozen in liquid nitrogen, and stored at −80 °C. The primers required for vector construction are listed in Table S1.

### Texture measurements

Fruit firmness was examined by a CTX texture analyser (AMETEK Brookfield, Middleborough, MA). The measurement points were located near the equator of the fruit and each fruit was measured at 4 points. The parameters were set as follows: probe diameter, 2 mm; penetration depth, 10 mm; descent speed, 1 mm/s; penetration speed, 1 mm/s; and post‐measurement speed, 10 mm/s.

### Determination of fibre composition

Cell wall material was extracted as described (Jin *et al*., 2016; Peng *et al*., 2000). And 0.3 g of plant material was ground in 1 mL of 80% ethanol and centrifuged to obtain the precipitate. After washing twice with 80% ethanol and acetone, the precipitate was the crude cell wall, then 1 mL of chloroform and methanol mixture (2:1, starch removed) was added and immersed for 15 h. The precipitate was centrifuged at 6000 ***g*** for 10 min at 25 °C, and the precipitate was washed twice with distilled water and dried (overnight at 70 °C) to obtain the cell wall material (CWM). Pectin, hemicelluloses, and cellulose fractions were determined as described previously (Cheng *et al*., 2019).

Cellulose extraction: Weigh about 5 mg CWM and add 0.5 mL distilled water. Slowly add 0.75 mL concentrated sulfuric acid and let stand for 30 min. Centrifuge for 10 min, take the supernatant, water bath at 95 °C for 10 min, absorb 1 mL in a glass colorimetric dish for determination of the light absorption value at 620 nm. Hemicellulose content was detected with hemicellulose content kit (Suzhou Keming Biotechnology Co., Ltd., Suzhou, China). Extraction of soluble pectin: Weigh 0.1 g CWM, add 2 mL distilled water, water bath at 50 °C for 30 min, take the supernatant, and set the volume to 10 mL. 0.1 mL of the extraction solution was taken, 0.6 mL of concentrated sulfuric acid was added, and then 0.2 mL 1.5 g/L carbazol–ethanol solution was added into the water bath at 95 °C for 20 min. After standing in the dark for 30 min, the absorbance of the reaction solution at 530 nm was measured.

### Gel tissue and cell wall material (CWM) swelling analysis

Equal amounts of WT and MdWRKY31‐OEs tomato fruit gel tissue from the B10 period were taken in 2 mL centrifuge tubes and centrifuged at 1000 ***g*** for 1 min to observe gel stratification.

CWM was extracted as described (Jin *et al*., 2016; Peng *et al*., 2000). An equal amount of CWM was taken in a 1.5 mL centrifuge tube, the toluidine blue was added to stain CMW and samples were placed at 25 °C for 24 h to precipitate. The height of stained CMW was measured to quantitate CWM swelling.

### Wax extraction and content determination

Tomato fruits were immersed in chloroform for 1 min twice with 25 μL of 10 mg/L n‐tetracosane as an internal standard (Zhang *et al*., 2023). The extracts were filtered using organic filter paper before evaporating to dryness at 50 °C under rotational evaporation, reconstituted into serum bottles with chloroform: methanol (10:1, v/v) (Kong *et al*., 2024). The chloroform: methanol (10:1, v/v) was then evaporated under gaseous N_2_, and the dried extract represents the total wax (μg). Stored in −20 °C refrigerators for later use (Kong *et al*., 2024; Zhang *et al*., 2023).

The surface area of the tomato was determined following the method of Ketata *et al*. (2013). Briefly, fruit's equatorial diameter *d*1, polar diameter *d*2, and high h were recorded using a digital vernier caliper. The surface area was calculated as *S* = 4π*r* ^2^, *r* = (*d*1 + *d*2 + *h*)/6. The amount of wax present was stated in micrograms per unit fruit surface area (μg/cm^2^) (Kong *et al*., 2024).

### Wax preparation and chemical analysis of testing condition with GC–MS

The waxes were re‐dissolved in 10 mL of chloroform:methanol (10:1, v/v) with internal standard of n‐tetracosane (10 mg/L). 1 mL of the sample was dried under a stream of nitrogen and then derived with 200 μL of bis‐N,N‐(trimethylsilyl) trifluoroacetamide (BSTFA; Sigma, St. Louis, MO) for 60 min at 70 °C. After removing BSTFA under a nitrogen flow, those derivatives were dissolved in 1 mL chloroform for GC–MS analysis (Belge *et al*., 2014; Vogg *et al*., 2004; Wang *et al*., 2014). The method refers to Zhang *et al*. (2023), and has been modified: Samples were injected out of chloroform in a gas chromatograph coupled to a mass spectrometer (ionization voltage: 10–200 eV; resolution: *R* > 2 M (RWHM); scanning speed: 12 500 u/s). The temperature program was as follows:1 min at 70 °C, increase from 70 to 200 °C at 10 °C/min, increase at 4 °C/min to 280 °C, and held at 280 °C for 25 min. Peaks were quantified based on their FID ion current and library NIST17.1 as described by Zhang *et al*. (2023).

### Scanning electron microscopy (SEM)

SEM was used to observe epicuticular wax. Apple peels were collected from tomato. Samples were vacuum‐dried at −45 °C in a 25‐Pa vacuum for 24 h DDU‐1110, 50/60 Hz, 1.7 kVA and viewed by cryo‐SEM as described by previous studies (Lü *et al*., 2009; Zhang *et al*., 2023).

### Statistical analysis

At least three biological replicates and three technical replicates were performed for each experiment, and each data point is the mean ± SD of the three replicates. Significant differences between normally distributed data were assessed by Student *t*‐test or one‐way analysis of variance (ANOVA) and SNK multiple comparison test. Differences with *P* < 0.05 were considered to be statistically significant.

## Conflict of interests

None declared.

## Author contributions

D.G.H. conceived, designed, and supervised the project. J.H.W., Q.S., C.N.M, M.M.W., and C.K.W. performed the experiments. J.H.W., Q.S., and D.G.H. wrote and revised the paper. J.H.W., Q.S., C.N.M., M.M.W., C.K.W., Y.W.Z., and W.Y.W. analysed the data. All authors discussed the results and commented on the manuscript.

## Supporting information


**Figure S1** Analysis of the cuticular wax of tomato fruit during postharvest storage.
**Figure S2** Real‐time quantitative PCR (RT‐qPCR) validation of selected cell wall‐modifying enzymes at MG, BR, B5, and B10 in tomato pericarp and gel of WT and *MdWRKY31* overexpression tomatoes.
**Figure S3** MdWRKY31 does not bind to the promoters of *MdPG1* and *MdEXPA3*.
**Figure S4** Analysis of *MdXTH2* promoter *cis*‐acting elements.
**Figure S5** The expression of genes that involved in cell wall‐modifying and ethylene biosynthesis in TRV and MdXTH2‐TRV apple fruit.
**Figure S6** Analysis of average fruit weight of tomato fruit during fruit ripening.
**Table S1** List of primers used in this study.

## Data Availability

The data that support the findings of this study are available from the corresponding author upon reasonable request. Sequence data from this article can be found in the GDR/GenBank data libraries and NCBI Database under the following accession numbers: MdWRKY31 (MD05G1349800); MdXTH2 (MD13G1237300); MdEXPA3 (MD12G1142900); MdPG1 (MD12G1077400); MdNAC7 (MD09G1006400); SlCEL2 (Solyc09g010210); SlEXP1 (Solyc06g051800); SlPG2a (Solyc10g080210); SlXTH5 (Solyc01g081060); SlPL1 (Solyc09g091430).
