# Supplementary material for: MdWRKY31‐MdNAC7 regulatory network: orchestrating fruit softening by modulating cell wall‐modifying enzyme MdXTH2 in response to ethylene signalling
Source: Plant Biotechnol J. 2024 Aug 23;22(12):3244–61. doi: 10.1111/pbi.14445 (PMC11606422; doi:10.1111/pbi.14445)
Supplement: Supplementary file 1 — Figure S1 Analysis of the cuticular wax of tomato fruit during postharvest storage. Figure S2 Real‐time quantitative PCR (RT‐qPCR) validation of selected cell wall‐modifying enzymes at MG, BR, B5, and B10 in tomato pericarp and gel of WT and MdWRKY31 overexpression tomatoes. Figure S3 MdWRKY31 does not bind to the promoters of MdPG1 and MdEXPA3. Figure S4 Analysis of MdXTH2 promoter cis‐acting elements. Figure S5 The expression of genes that involved in cell wall‐modifying and ethylene biosynthesis in TRV and MdXTH2‐TRV apple fruit. Figure S6 Analysis of average fruit weight of tomato fruit during fruit ripening. Table S1 List of primers used in this study. [file PBI-22-3244-s001.docx]

**Plant Biotechnology Journal Supporting Information**

**Running Title:** MdWRKY31-MdNAC7 module dominates fruit softening.

**The Title:** MdWRKY31-MdNAC7 regulatory network: orchestrating fruit

softening by modulating cell wall-modifying enzyme MdXTH2 in response to

ethylene signaling

**The full names of all the authors:**

Jia-Hui Wang1,2#, Quan Sun1#, Chang-Ning Ma1, Meng-Meng Wei1, Chu-Kun

Wang1, Yu-Wen Zhao1, Wen-Yan Wang1, Da-Gang Hu1*

**The names and address of the institution:**

1National Key Laboratory of Crop Biology, Shandong Collaborative Innovation

Center of Fruit & Vegetable Quality and Efficient Production, College of

Horticultural Science and Engineering, Shandong Agricultural University, Tai’an,

Shandong 271018, China

2College of Horticulture, Agricultural University of Hebei, Baoding, Hebei,

071000, China

**Corresponding authors:**

Da-Gang Hu (fap_296566@163.com)

Tel: +86-538-824-6151; Fax number: +86-538-824-2364

Address: College of Horticulture Science and Engineering, Shandong

Agricultural University, Taian, Shandong 271018, China

**One-sentence summary:** The MdWRKY31-MdNAC7 complex emerges as a

key regulatory hub connecting ethylene signaling with downstream gene

expression, specifically *MdXTH2*, a critical player in apple fruit softening.

**Note:** #These authors contributed equally to this work; *Correspondence:

fap_296566@163.com


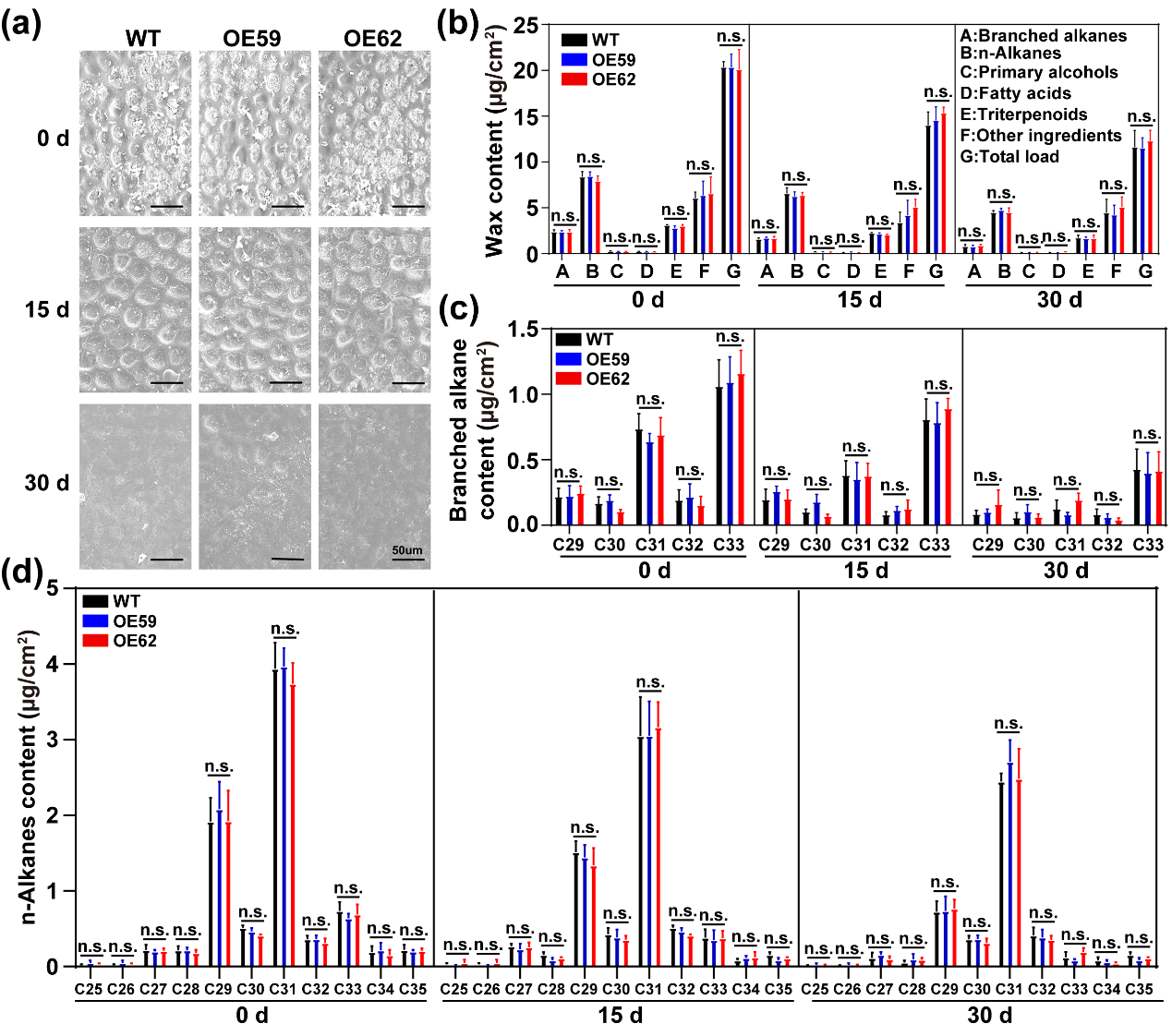


**Figure S1.Analysis of the cuticular wax oftomato fruit during postharvest**

**storage.**

(a) Cuticular wax crystals of tomato fruit. Bars=50µm. (b) Analysis of cuticular

total wax content and wax component content. (c) Branched alkane

composition and content. (d) n-Alkane composition and content. Statistically

significant differences determined by Student’s t-test (n.s., no significant

difference).


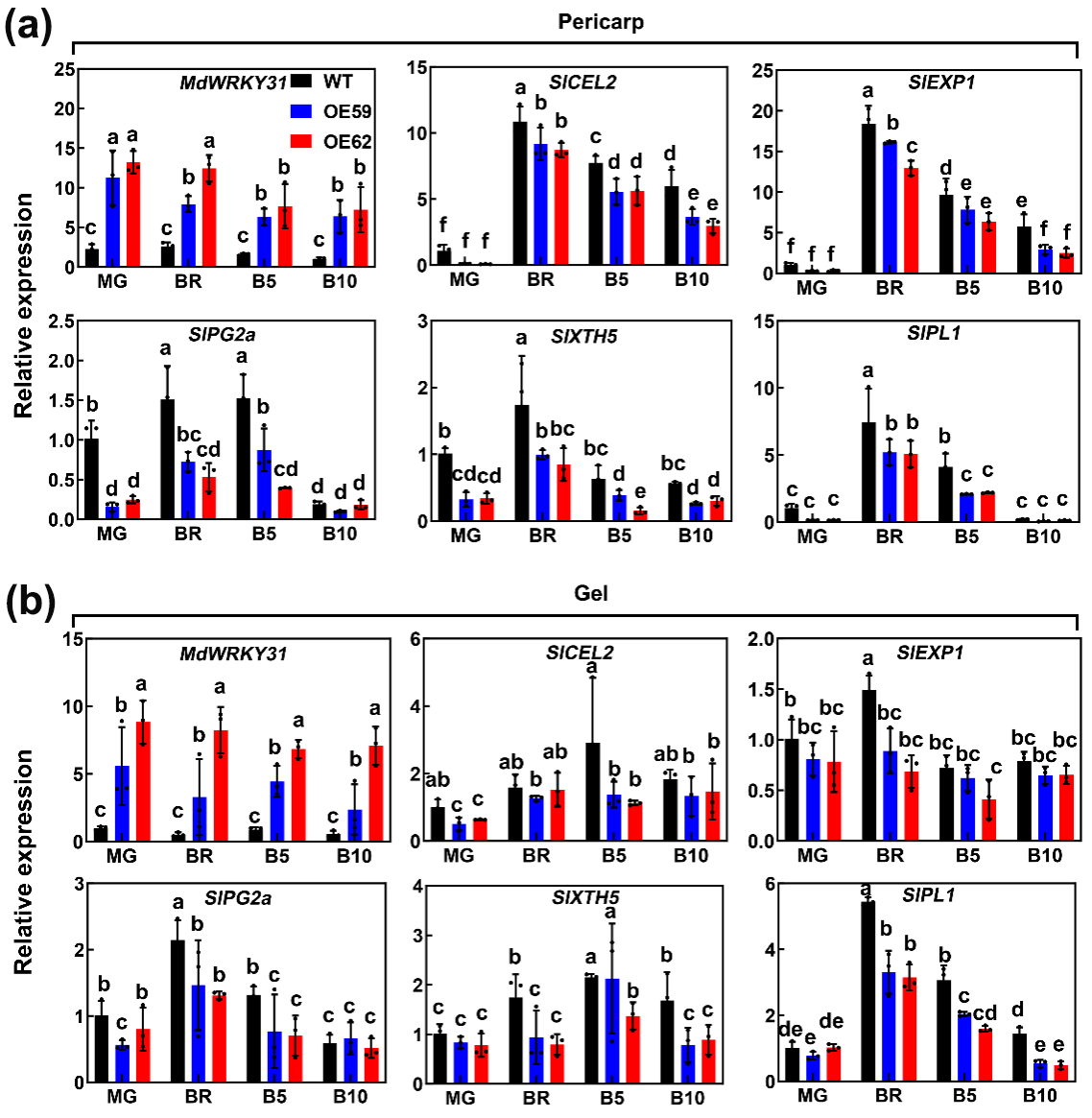


**Figure S2. Real-time quantitative PCR (RT-qPCR) validation of selected**

**cell wall-modifying enzymes at MG, BR, B5, and B10 in tomato pericarp**

**and gel of WT and *MdWRKY31* overexpression tomatoes.**

RT-qPCR for the expression of cell wall-modifying enzymes in tomato pericarp

**(a)**and gel**(b)**at MG, BR, B5, andB10. MG (mature green stage, approximately

39 days after flowering), BR (breaker stage, when fruit change from green to

yellow-brown, approximately 42 days after flowering), B5 (light red,

approximately breaker + 5 days), and B10 (full red stage, approximately

breaker + 10 days). Every experiment was performed independently and

repeated at least three times. Error bars indicate SD. Different letters above the

columns indicate significant differences (*P* < 0.05) as determined by one-way

ANOVA


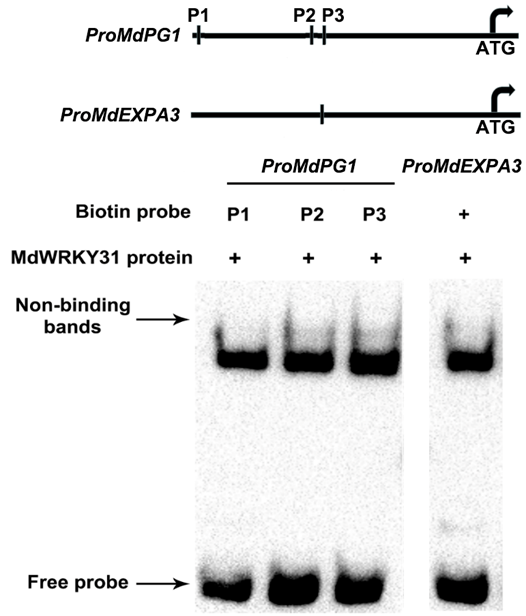


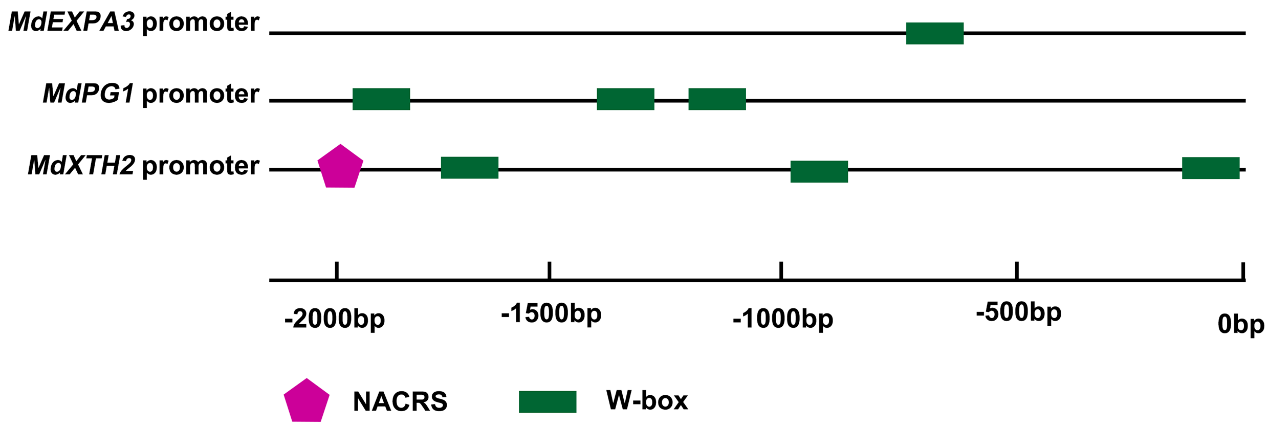


**Figure S3. MdWRKY31 does not bind to the promoters of *MdPG1* and *MdEXPA3*.**

No binding bands were detected in EMSA after co-incubation of MdWRKY31 protein with the biotin probe of *MdPG1* or *MdEXPA3*.

**Figure S4. Analysis of *MdXTH2* promoter *cis*-acting elements.**


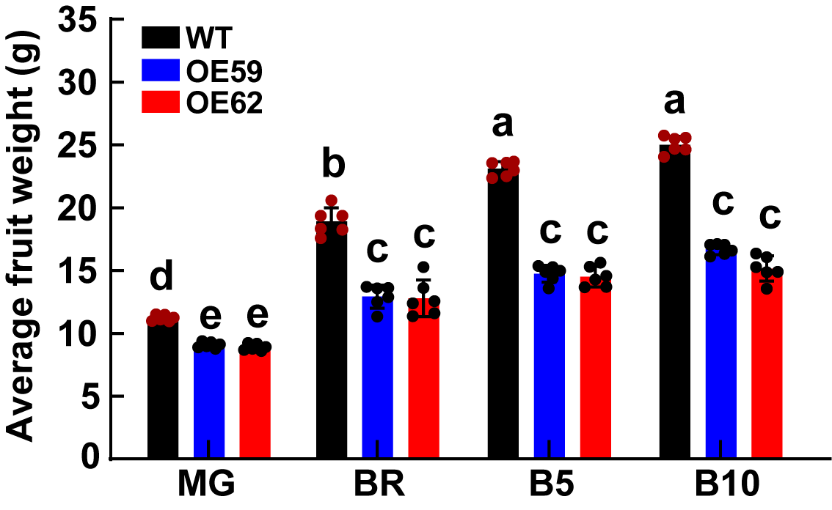

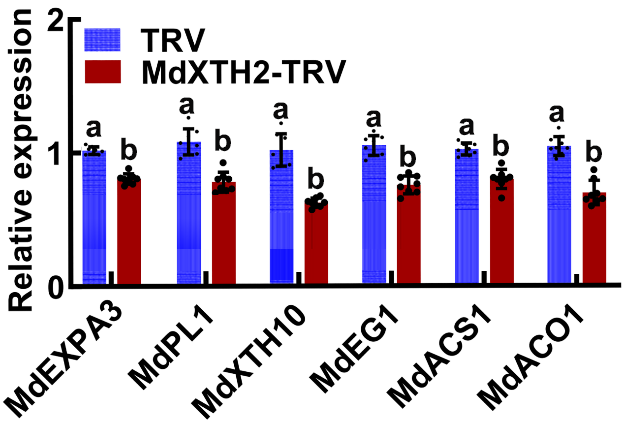


**Figure S5. The expression of genes that involved in cell wall-modifying**

**and ethylene biosynthesis in TRV and MdXTH2-TRV apple fruit.**

For each gene analysis, eight biological and three technical repetitions were

performed. Data are shown as the mean ± SD. Different letters above the

columns indicate significant differences (*P* < 0.05) as determined by one-way

ANOVA.

**Figure S6. Analysis of average fruit weight of tomato fruit during fruit**

**ripening.**

OE59 and OE62 were *MdWRKY31*-overexpressing fruit lines, respectively.

Wild-type (WT) was used as control. Data are shown as the mean ± SD.

Experiments were repeated nine independent times. Different letters above the

columns indicate significant differences (*P* < 0.05) as determined by one-way

ANOVA.

**Table S1. List of primers used in this study.**

**Name**

18S-F

18S-R

MdXTH2-F (qRT-PCR)

MdXTH2-R (qRT-PCR)

MdXTH7-F (qRT-PCR)

MdXTH7-R (qRT-PCR)

MdAFase-F (qRT-PCR)

MdAFase-R (qRT-PCR)

MdEG1-F (qRT-PCR)

MdEG1-R (qRT-PCR)

MdEXPA3-F (qRT-PCR)

MdEXPA3-R (qRT-PCR)

MdEXPA7-F (qRT-PCR)

MdEXPA7-R (qRT-PCR)

MdPLA18-F (qRT-PCR)

MdPLA18-R (qRT-PCR)

MdPL-F (qRT-PCR)

MdPL-R (qRT-PCR)

MdNA7-F (qRT-PCR)

MdNA7-R (qRT-PCR)

SlAP2a-F (qRT-PCR)

SlAP2a-R (qRT-PCR)

SlXTH5-F (qRT-PCR)

SlXTH5-R (qRT-PCR)

SlPG2a-F (qRT-PCR)

SlPG2a-R (qRT-PCR)

**Primer sequences**

TGACCGAATGAGCAAGGAAATTACT

TACTCAGCTTTGGCAATCCACATC

GAACCAACACTGCAGCACTG

CAAAGTGCTGGAGAGGAGGG

TCAGCTGATGATGCAAGGCA

ACCCGCCTCAATGTCGAAAT

TACTACGACCCGAGAAGCCA

CGCATTTCCGTTGCCGTAAT

GGATCTGGTGGGAGGGTACT

CAGTGTTCCCATCTCCCACC

TTCCCCTACCTCCCCAAAAG

GACCCGCCATGAAAAACCCAGATGAAGA

TCTTCTTCTTCACCACCGCC

GGGAGATGAGGTCCTGGACT

GTCTCCTAACCCTACCCCTTAC

GAATGAGGTTTGGAAAAAACA

ATCCGACTTGCAAGCCTCAA

TGAATCTGCCGGGCGTATAC

AATCCGGAAACTGGGTGGTC

TCGGCATGCTAACACTGTGT AGAAATGGGGGACAATAGGG

ATTGTTGCTGCTCGGAGTCT

ATTCAGCCATCTCTTTGGTGAT

ACTTGAACCCTGAACCTGTGTT

AGCTAAGGGTGATGGAAAAACA

TGAAAAGGTGATTTGCTTGAGA

SlE4-F (qRT-PCR)

SlE4-R (qRT-PCR)

SlE8-F (qRT-PCR)

SlE8-R (qRT-PCR)

SlAP2a-F (qRT-PCR)

MdWRKY31-F (AD)

MdWRKY31-R (AD)

MdNAC7-F (PGEX)

MdNAC7-R (PGEX)

pMdXTH2-F

pMdXTH2-R

MdWRKY31-F(PET32a)

MdWRKY31-R(PET32a)

MdNAC7-F (PGEX)

MdNAC7-R (PGEX)

MdWRKY31-F (62SK)

MdWRKY31-R (62SK)

MdXTH2-F (TRV)

MdXTH2-R (TRV)

MdWRKY31-F (TRV)

MdWRKY31-R (TRV)

MdNAC7-F (TRV)

MdNAC7-R (TRV)

MdNAC7-F (pIR)

MdNAC7-R (pIR)

TCTAAATCGCCAGGGTAATGAT

TAGCTTCTAACGACTCCCTTGC

TTACATGGCTCCGAATCCTC

CGAGACCGAGACCTTCAGAC

AGAAATGGGGGACAATAGGG

GGATCCATGTGTGGTGGTGCTATCATTTCCG

GTCGACATACAGAAGCTGCCCTTGTTGCTG

GAATCTGAGGTGGACCGTGCTTAC

CCGCCATCGCTTTGGACTTCTC

GAATTCCCGGGATTTTTCAGTGTGATCGGCAT

TTCGCGAACGCGTGAGCTCATTAGAAGGATAT

GTCGACATGGACAAAGGATGGGGGCTC

AAGCTTATTTCCCGGGAAGCTGCTAATGTT

GAATCTGAGGTGGACCGTGCTTAC

CCGCCATCGCTTTGGACTTCTC

TCTAGAATGGACAAAGGATGGGGGCTC

GTCGACATTTCCCGGGAAGCTGCTAATGTT

GGATCCCTGCATATCAATTTTTCCGA

CCCGGGATGACTAAGATATTCCTTGTT

GCATCTTGCTGCAGTTGTA

CAACTCTTTTCCCGTCACG

GAATTCAATATTAATCGGCATGCT

CCCGGGCAAAGAACATCAGATGAT

AAGCTTATGGAGAATATTAAAG

TCTAGAAATATTAATCGGCATGC
